# Histone Deacetylases Suppress CGG Repeat–Induced Neurodegeneration Via Transcriptional Silencing in Models of Fragile X Tremor Ataxia Syndrome

**DOI:** 10.1371/journal.pgen.1001240

**Published:** 2010-12-09

**Authors:** Peter K. Todd, Seok Yoon Oh, Amy Krans, Udai B. Pandey, Nicholas A. Di Prospero, Kyung-Tai Min, J. Paul Taylor, Henry L. Paulson

**Affiliations:** 1 Department of Neurology, University of Michigan, Ann Arbor, Michigan, United States of America; 2 Department of Genetics, Louisiana State University Health Sciences Center, New Orleans, Louisiana, United States of America; 3 Neurogenetics Branch, National Institute of Neurological Disorders and Stroke, Bethesda, Maryland, United States of America; 4 Department of Biology, University of Indiana, Bloomington, Indiana, United States of America; 5 Department of Developmental Neurobiology, St. Jude Children's Research Hospital, Memphis, Tennessee, United States of America; The Hospital for Sick Children and University of Toronto, Canada

## Abstract

Fragile X Tremor Ataxia Syndrome (FXTAS) is a common inherited neurodegenerative disorder caused by expansion of a CGG trinucleotide repeat in the 5′UTR of the fragile X syndrome (FXS) gene, *FMR1*. The expanded CGG repeat is thought to induce toxicity as RNA, and in FXTAS patients mRNA levels for FMR1 are markedly increased. Despite the critical role of FMR1 mRNA in disease pathogenesis, the basis for the increase in FMR1 mRNA expression is unknown. Here we show that overexpressing any of three histone deacetylases (HDACs 3, 6, or 11) suppresses CGG repeat–induced neurodegeneration in a *Drosophila* model of FXTAS. This suppression results from selective transcriptional repression of the CGG repeat–containing transgene. These findings led us to evaluate the acetylation state of histones at the human *FMR1* locus. In patient-derived lymphoblasts and fibroblasts, we determined by chromatin immunoprecipitation that there is increased acetylation of histones at the *FMR1* locus in pre-mutation carriers compared to control or FXS derived cell lines. These epigenetic changes correlate with elevated FMR1 mRNA expression in pre-mutation cell lines. Consistent with this finding, histone acetyltransferase (HAT) inhibitors repress FMR1 mRNA expression to control levels in pre-mutation carrier cell lines and extend lifespan in CGG repeat–expressing *Drosophila*. These findings support a disease model whereby the CGG repeat expansion in FXTAS promotes chromatin remodeling in *cis*, which in turn increases expression of the toxic FMR1 mRNA. Moreover, these results provide proof of principle that HAT inhibitors or HDAC activators might be used to selectively repress transcription at the *FMR1* locus.

## Author Summary

FXTAS is a common inherited neurodegenerative disorder resulting from accumulation of a toxic CGG repeat–containing mRNA species in the brain. For unknown reasons, expression of this toxic mRNA is markedly increased in patients, and this increase is thought to contribute to pathogenesis. Here we used a fruit fly model of FXTAS and patient-derived cells to investigate the cause of increased toxic RNA production in this disorder. We identified histone deacetylases (HDACs) as genetic suppressors of the neurodegenerative phenotype in *Drosophila*. These HDAC modifiers rescue the phenotype by selectively lowering expression of the toxic mRNA. These findings led us to evaluate the acetylation state of histones at the human *FMR1* locus. We found increases in histone acetylation surrounding the CGG repeat in pre-mutation carriers. These changes were associated with increased transcription of FMR1 RNA. Moreover, we were able to reverse these changes and lower production of the toxic mRNA with drugs that inhibit histone acetylation. These same drugs also extended lifespan in FXTAS model flies. Taken together, our studies suggest a novel mechanism by which FMR1 mRNA transcription is increased in FXTAS, and they provide a proof of principle that such changes are dynamic and modifiable by genetic or pharmacologic alterations.

## Introduction

Fragile X tremor ataxia syndrome (FXTAS) is a recently described adult onset neurodegenerative disorder affecting approximately 1∶3000 men and, less frequently, women over the age of 50[1]. Affected individuals present with slowly progressive gait ataxia, intention tremor, dementia, parkinsonism and neuropsychiatric symptoms[2]. Pathologically, FXTAS patients develop cerebellar and cortical atrophy with widespread neurodegeneration. These gross pathologic changes are associated with intranuclear ubiquitin-positive inclusions in neurons and astrocytes of the cerebellum and cerebral cortex [3], [4].

FXTAS results from pathological expansion of a CGG trinucleotide repeat in the 5′UTR of the *FMR1* gene. Normal repeats are less than 55 CGGs. Expansion to greater than 200 CGGs leads to transcriptional silencing of *FMR1*, causing Fragile X Syndrome, a common inherited cause of mental retardation. By contrast, FXTAS patients have modest expansions of between 55 and 200 CGG repeats. Intriguingly, this “pre-mutation” repeat FMR1 mRNA is transcribed at 2–10 fold greater levels than in control patients[5]. Because the expanded CGG repeat is inefficiently translated, however, FMR protein (FMRP) levels are normal or decreased in FXTAS patient-derived cell lines and in animal models of the disorder [5]–[12].

Compelling evidence indicates that the expanded CGG repeat-containing mRNA is the primary toxic species in FXTAS. Expression of an expanded CGG repeat sequence in the 5′UTR of eGFP in human cell lines, *Drosophila*, or mouse Purkinje neurons is sufficient to elicit toxicity[13]–[16]. A leading hypothesis for disease pathogenesis is that FMR1 mRNA containing an expanded CGG repeat binds to and sequesters important proteins within nuclear inclusions, preventing them from performing their normal functions [17]–[20].

Because FXTAS is thought to result from an RNA toxic gain-of-function, the accumulation of increased FMR1 mRNA is likely an important and proximal event in pathogenesis. However, work to date has failed to identify the critical events driving this increase in toxic mRNA. At least three non-exclusive mechanisms exist by which FMR1 mRNA accumulation could be altered. There could be increased transcription at the *FMR1* locus via a feedback loop based on inefficient FMRP translation; presumably this would be mediated via activation of a specific transcription factor cascade. Evidence against this mechanism includes normal *FMR1* mRNA levels in a patient with a deleterious point mutation in FMRP[21] and in patients with very large unmethylated CGG repeats who translate little or no protein[22]–[24]. Alternatively, there could be increased mRNA stability as a result of the altered secondary structure of the FMR1 message. However, reports to date suggest that excess transcription rather than altered mRNA stability is critical to the accumulation of FMR1 mRNA [5], [25]. A third possibility, which to date has only been explored *in vitro* [26], [27], is that the premutation range CGG repeat itself could lead to alterations in the local DNA and/or chromatin structure at the *FMR1* locus, stimulating increased basal transcription in *cis*.

Here we sought to determine the cause of increased transcription in premutation carriers using *Drosophila* and cell-based model systems. Our results provide evidence both that the expanded CGG repeat enhances its own transcription in *cis* via alterations in local chromatin structure and that this transcriptional augmentation may be pharmacologically modifiable.

## Results

**Figure 1:**
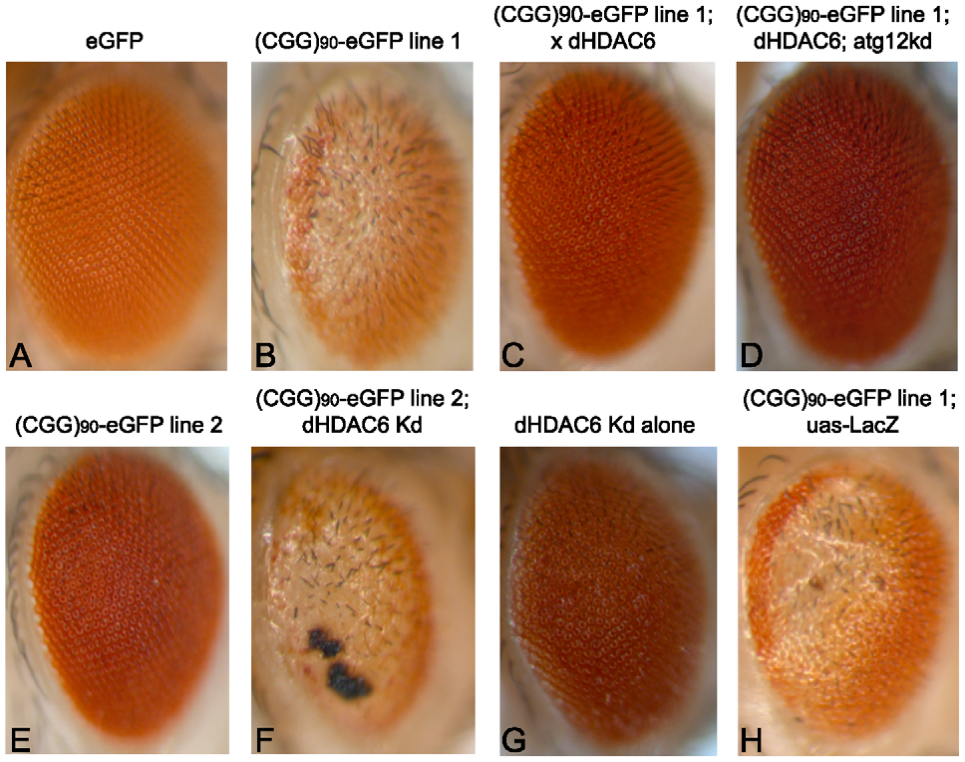
HDAC6 suppresses (CGG)_90_-eGFP–induced neurodegeneration by an autophagy independent mechanism. Expression of eGFP alone in the fly eye with a gmr-GAL4 driver has no notable phenotype (A). When a CGG repeat is placed in the 5′UTR of eGFP, a dose- and temperature-dependent rough eye phenotype develops that is more severe in line 1 (B) than line 2 (E). The severe rough eye phenotype is suppressed by co-expression of HDAC6(C). This phenotypic rescue by HDAC6 does not depend on an intact autophagy pathway, as siRNA-based knockdown of atg12 does not prevent suppression (D). The mild rough eye phenotype seen in line 2 is synergistically enhanced by siRNA-based knockdown of dHDAC6, producing a black necrotic eschar on portions of the eye(F). Of note, dHDAC6 knockdown induces a mild rough eye phenotype when expressed alone (G). As a negative control, expression of Beta-Gal (lacZ) has no effect on the phenotype. (H). All images are representative of >100 flies per genotype and at least two separate crosses. KD  =  knock down.

To better understand the pathophysiology of FXTAS, we performed a screen of candidate genetic modifiers in an established *Drosophila* model of CGG-repeat induced neurodegeneration, testing known modifiers of other neurodegenerative disease models. As previously described [13], expression of an expanded CGG repeat sequence (90 CGGs with two AGG interruptions) in the 5′ untranslated region of a heterologous transcript (enhanced Green Fluorescent Protein, eGFP) in the fly eye leads to a rough eye phenotype characterized by loss of pigmentation, omatidial disorganization and abnormal eye bristle patterning (Figure 1E versus Figure 1A)[13]. In lines expressing the transgene at higher levels, the rough eye is more severe, with loss of normal oomatidia formation and frank necrosis, especially when flies are reared at higher temperatures (Figure 1B versus Figure 1A, 1E).

One known modifier of polyglutamine toxicity in *Drosophila* is histone deacetylase 6 (dHDAC6). dHDAC6 is a Class 2B histone deacetylase and one of only two Class 2 HDACs in *Drosophila*. It has two human homologues: HDAC6 and HDAC10. HDAC6 deacetylates histones *in vitro*, and in *Drosophila* it acts on chromatin to influence the expression of hundreds of genes[28]. However, in mammalian systems it functions predominantly in the cytoplasm where it deacetylates multiple substrates including α-tubulin, cortactin and HSP90, and facilitates lysosomal degradation of ubiquitinated proteins (see [29] for a recent review). Most relevant to the current study, overexpression of dHDAC6 can rescue polyglutamine induced neurodegeneration in an autophagy-dependent manner in a *Drosophila* model [30].

**Figure 2:**
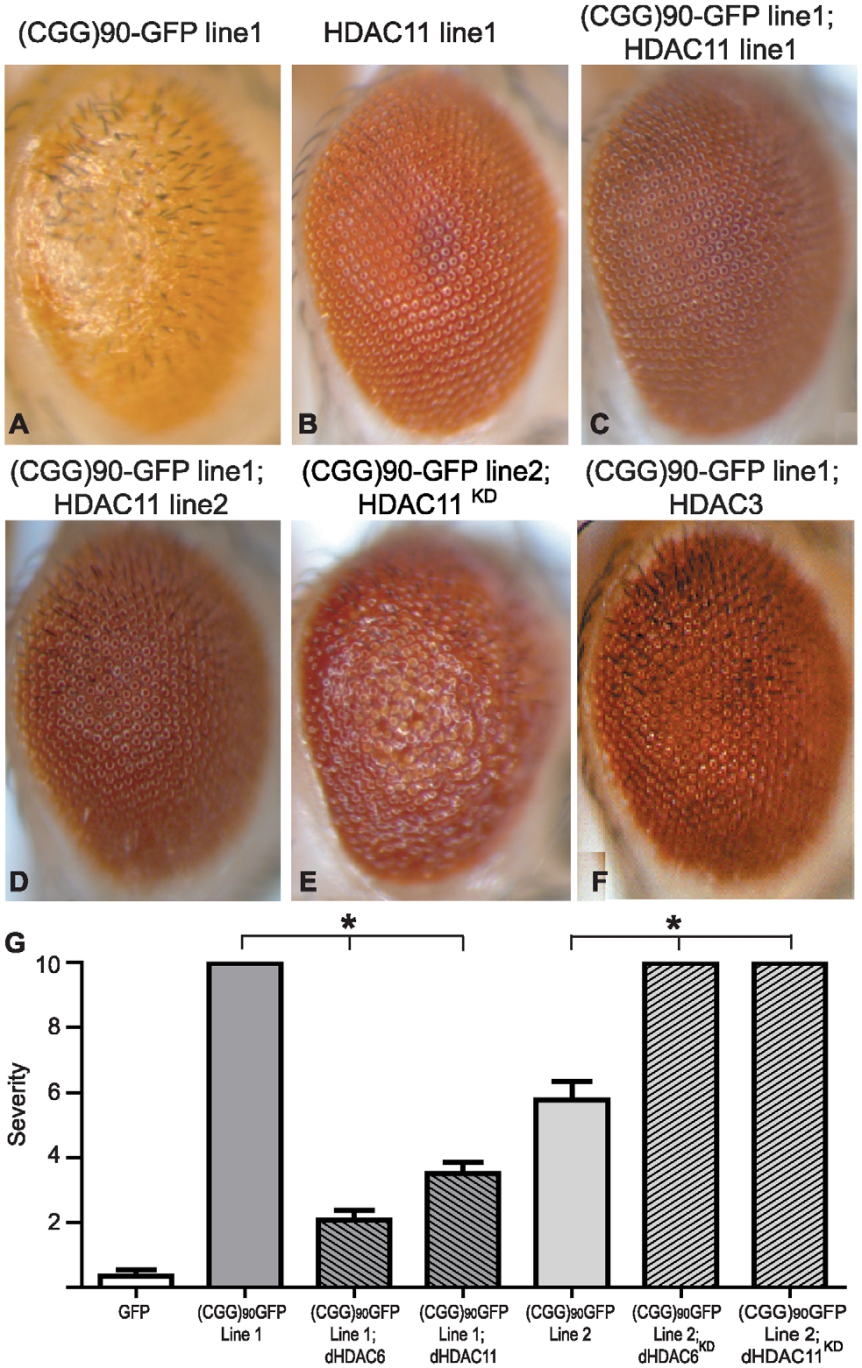
HDAC3 and HDAC11 also suppress (CGG)_90_-eGFP mRNA–induced neurodegeneration. Co-expression of HDAC11, a class IV HDAC and HDAC6 interacting partner, suppresses (CGG)_90_-eGFP induced neurodegeneration similarly to HDAC6 in either of two HDAC11-expressing lines (C and D compared to A). Overexpression of HDAC11 alone had no phenotype (B). Lowering endogenous dHDAC11 by siRNA significantly enhances (CGG)_90_-eGFP induced neurodegeneration (2E versus 1E). Similarly, overexpression of HDAC3, a class I HDAC, suppresses the (CGG)_90_-eGFP phenotype (F). G) Quantitation of the phenotypes in Figure 1 and Figure 2. Analysis of ∼20 flies per genotype demonstrates highly significant alterations in the phenotype. * represents P<0.01 by a Students' unpaired t-test. The lack of error bars on (CGG)_90_-eGFP line 1 and (CGG)_90_-eGFP line 2 co-expressed with siRNAs against HDAC6 and 11 reflect the fact that all observed flies had the most severe phenotype score.

We therefore were interested in assessing the influence of dHDAC6 activity in a *Drosophila* model of expanded CGG-related toxicity. Overexpression of dHDAC6 in FXTAS flies strongly suppressed the rough eye phenotype (Figure 1C, quantified in Figure 2G). Conversely, siRNA knockdown of dHDAC6 enhanced the CGG repeat induced phenotype (Figure 1F compared to Figure 1E and 1G, note that the black material on the eye in Figure 1F is eschar from necrosis). To test whether suppression of the CGG rough eye phenotype by dHDAC6 required activation of autophagy pathways, we utilized a fly line that co-expressed siRNA directed against the autophagy related protein 12 (atg12), a protein required for appropriate autophagic degradation of proteins. In contrast to dHDAC6 modification of polyglutamine-related toxicity, siRNA knockdown of Atg12 had no effect on suppression of the CGG repeat phenotype by dHDAC6 (Figure 1D). Co-expression of a control protein, beta-Gal, had no effect on the observed phenotype (Figure 1H) and expression of eGFP at comparable protein levels but without a CGG repeat in the mRNA had no intrinsic phenotype (Figure 1A).

Given that siRNA knockdown of autophagy pathways failed to inhibit dHDAC6 dependent rescue of CGG repeat toxicity, we hypothesized that dHDAC6 might be acting instead by altering transcription of the (CGG)_90_-eGFP repeat containing transgene. To test this idea, we first evaluated the effect of co-expression of other HDACs on CGG repeat induced neurodegeneration. Overexpression of either dHDAC3 (a major *Drosophila* class I HDAC, Figure 2F) or dHDAC11 (a novel class IV HDAC, Figure 2C and 2D, quantified in Figure 2G) also suppressed the CGG repeat-dependent rough eye phenotype. Conversely, siRNA directed against dHDAC11 exacerbated the CGG repeat phenotype (Figure 2E compared to Figure 1E). This differs from the observation in a *Drosophila* model of the polyglutamine disease SBMA, in which the phenotype is not modified by altering expression of dHDAC11 or dHDAC3 [30].

To further confirm a role for HDAC activity in modifying the CGG repeat dependent phenotype, we reared flies expressing eGFP or (CGG)_90_-eGFP on food containing either DMSO or the broad spectrum HDAC inhibitor SAHA. At doses known to suppress polyglutamine-induced neurodegeneration [31], the drug had no effect on the normal eye phenotype in eGFP expressing flies (Figure S1A). In contrast, (CGG)_90_-eGFP flies demonstrated a significant exacerbation of the rough eye phenotype in the presence of SAHA (Figure S1). In addition, SAHA partially suppressed the phenotypic rescue seen in flies co-expressing dHDAC6 and (CGG)_90_-eGFP (Figure S2), consistent with the phenotypic rescue being dependent on deacetylase activity. The lack of complete suppression of phenotypic rescue may be explained by the relative insensitivity of dHDAC6 to broad spectrum HDAC inhibitors [28], [32].

**Figure 3:**
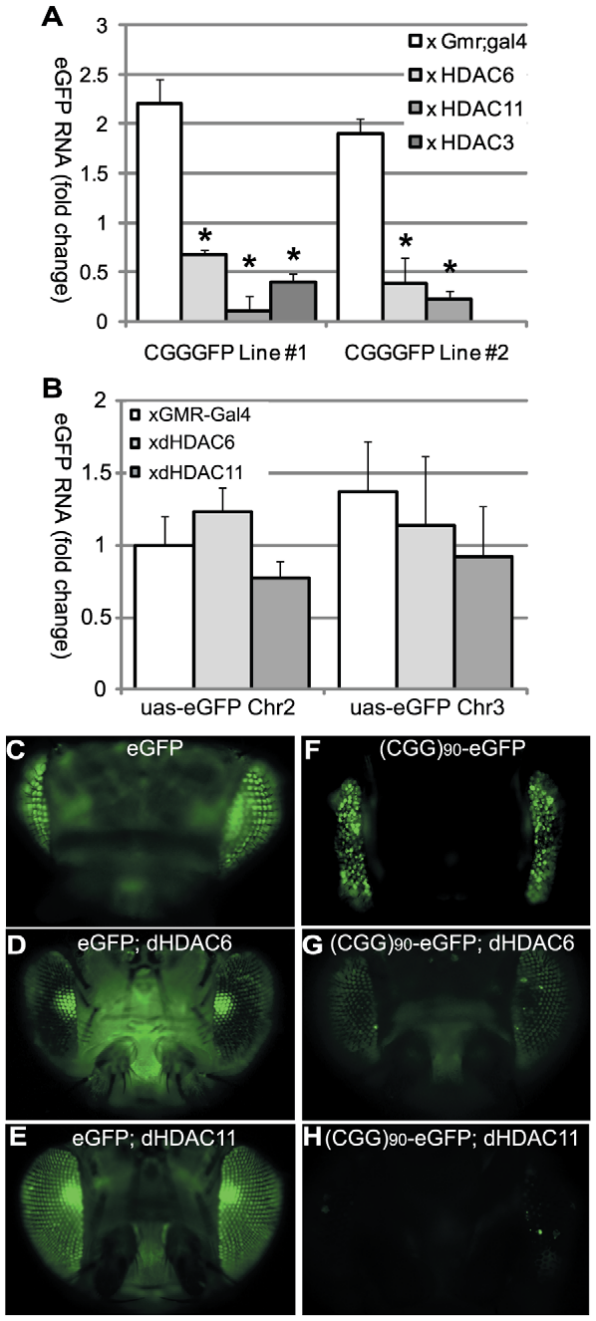
HDAC over-expression suppresses the accumulation of (CGG)_90_-eGFP mRNA. A) RT-PCR performed on total RNA isolated from the heads of flies with the noted genotypes. eGFP mRNA expression was normalized to18S RNA and expressed as fold change compared to uas-eGFP (Chr2) x gmr-Gal4 alone. (CGG)_90_-eGFP transcript levels for both line 1 and line 2 are significantly decreased when any of 3 classes of HDACs is co-expressed, compared to either line of (CGG)_90_-eGFP crossed with the gmr-Gal4 driver line alone. B) In contrast, there are no significant changes in eGFP mRNA levels when HDAC6 or HDAC11 is co-expressed with either of 2 uas–eGFP lines with different chromosomal insertion sites. Data represent the summary of 3 independent experiments with n>10 for each genotype. (C–H) eGFP protein expression in fly lines was visualized by fluorescence. Co-expression of HDAC6 (D) or HDAC11 (E) with eGFP leads to little or no decrease in fluorescent signal. In contrast, eGFP protein expression in (CGG)_90_-eGFP flies (F) is markedly reduced by co-expression of HDAC6 (G) or HDAC11 (H). * = P<0.01 versus (CGG)_90_-eGFP alone by a Students unpaired t-test.

If overexpression of HDACs leads to phenotypic rescue by reducing histone acetylation, the mRNA levels of the transgene correspondingly would be expected to be lower in (CGG)_90_-eGFP flies co-expressing HDACs. We therefore evaluated eGFP mRNA levels in (CGG)_90_-eGFP flies at baseline and when HDACs were modulated. Overexpression of HDAC3, HDAC6 or HDAC11 reduced (CGG)_90_-eGFP mRNA in Line 1 flies and HDAC6 or HDAC11 reduced (CGG)_90_-eGFP mRNA in Line 2 flies as measured by real-time semi-quantitative RT-PCR (Figure 3A). In contrast, overexpression of dHDAC6 or dHDAC11 did not cause changes in eGFP transcript levels in the absence of the CGG repeat in two separate uas-eGFP fly lines with different chromosomal insertion sites, consistent with previously published results[33] (Figure 3B). Similarly, eGFP protein expression was selectively decreased when (CGG)_90_-eGFP fly lines, but not eGFP lines, co-expressed HDAC6 or 11 (Figure 3F–3H versus Figure 3C–3E and additional data not shown). These findings suggest that transcriptional repression of these transgenes is dependent on the presence of the expanded CGG repeat sequence. It is worth noting that these three HDAC proteins do not alter expression of other transgenes, such as a polyglutamine-containing androgen receptor in a *Drosophila* model of SBMA[30] or uas-alpha synuclein in a *Drosophila* model of Parkinson's Disease[33]. Moreover, global HDAC inhibition or selective siRNA knockdown of HDAC3 or 6 does not alter expression of uas-HTT transgenes in a separate *Drosophila* model of polyglutamine disease [34].

**Figure 4:**
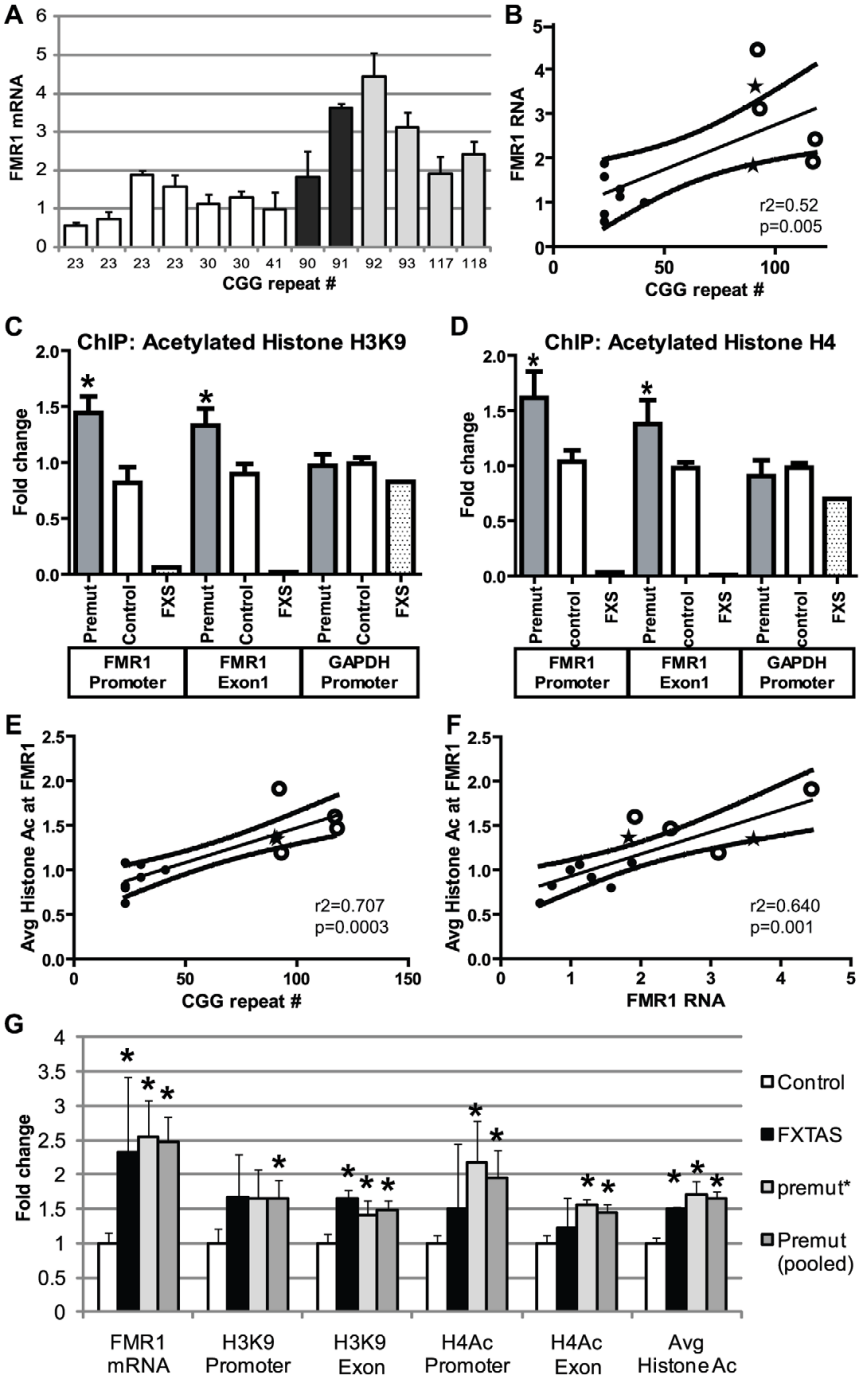
Chromatin alterations at *FMR1* locus in pre-mutation expansion carrier lymphoblasts. A) FMR1 mRNA expression by qRT-PCR from 13 lymphoblastoid cell lines derived from male patients in fragile X families. FMR1 mRNA levels, normalized to actin mRNA, are expressed relative to a control cell line included in all experiments ((CGG)_41_). White bars =  control cell lines. Black bars =  confirmed FXTAS cases. Light grey bars =  pre-mutation carriers whose clinical status is unknown. B) Correlation between CGG repeat size and FMR1 mRNA. Solid black dots =  control cell lines, stars =  confirmed FXTAS cases, open circles =  pre-mutation carriers whose clinical status is unknown. The central line is the linear best fit. Curved dashed lines are 95% confidence intervals. The correlation is significant (Spearman correlation r^2^ = 0.515, p = 0.005). C) ChIP against acetylated Histone H3K9 from all pre-mutation carriers (FXTAS and unknown clinical status, dark gray bars), controls (white bars), or FXS (spotted bars, 1 cell line) patient-derived cell lines. PCR primers were targeted to the *FMR1* promoter, the first exon of *FMR1*, or the GAPDH promoter as a control. D) ChIP against acetylated Histone H4. For (C) and (D), ChIP DNA expression is normalized to input DNA and then graphed as fold change from a control cell line included in all experiments. E) Correlation between CGG repeat length and H3/H4 acetylation status. Repeat length plotted against mean fold change for association of the *FMR1* locus with H3K9 or H4 acetylated histones. A combination of both markers at both the promoter and exon provided the best fit model. F) Correlation between H3/H4 histone acetylation and FMR1 mRNA levels. G) Comparison of confirmed FXTAS cases (black bars, n = 2) and pre-mutation lines whose clinical status is unknown (light grey bars, n = 4) with controls (white bars, n = 7). Dark grey bars =  Pooled data on all pre-mutation lines (n = 6). * = P<0.05 t-test vs controls.

These initial experiments were performed in *Drosophila* in the absence of the full endogenous human promoter and chromatin context. To extend our results to the human genomic context, we analyzed patient-derived lymphoblast cell lines. First, we confirmed a previous report [5] that FMR1 mRNA levels are elevated in lymphoblasts derived from patients with expanded (>80) CGG repeats compared to lymphoblasts from normal controls (<45 CGG repeats) (Figure 4A, 4B). CGG repeat length correlated with mRNA expression (r^2^ = 0.458, p = 0.01, Figure 4C). Two of the pre-mutation lymphoblast cell lines (#C0051.004, (CGG)_90_, and #C014.004, (CGG)_91_, a kind gift from Stephanie Sherman) were derived from patients with clinically probable FXTAS. Four other pre-mutation carrier cell lines were obtained from the Coriell cell repository; clinical information on these cases is not available. There was no difference in mRNA expression between the clinically confirmed FXTAS cases and the Coriell derived lines (Figure 4A and 4G). We therefore grouped data from all the pre-mutation carrier cell lines for analysis. Individual profiles of each cell line are presented in Figure S4.

Next we investigated chromatin modifications at the *FMR1* locus surrounding the CGG repeat by performing chromatin immunoprecipitation (ChIP) on lymphoblasts using antibodies that recognize acetylated histones associated with active chromatin[35]. In both control and pre-mutation carrier cell lines, we observed specific pulldown of the *FMR1* locus with antibodies directed against either acetylated histone H3K9 or pan-acetylated histone H4 compared to non-specific antibodies (Figure S3).

To investigate whether the expanded CGG repeat in pre-mutation carriers influences the acetylation state of histones, we compared the degree of pull down for regions of the *FMR1* locus directly surrounding the CGG repeat (the proximal *FMR1* promoter and in the first exon of *FMR1*) by using semi-quantitative real time PCR on DNA derived from the immunoprecipitates. ChIP of acetylated histone H3K9 demonstrated significantly elevated association of the *FMR1* locus surrounding the CGG repeats in pre-mutation patient derived cell lines compared to control individuals with normal CGG repeat sizes (Figure 4D). Similar results were obtained when ChIP was performed with antibodies directed against pan-acetylated Histone H4 (Figure 4E). As previously described, the *FMR1* locus in a single cell line derived from a Fragile X Syndrome patient with a highly expanded and methylated CGG repeat showed little association with acetylated histones (Figure 4D, 4E) [36]–[38]. As a control, primers directed against the promoter region for GAPDH, which is known to associate preferentially with acetylated histones, did not detect any significant differences in expression between control and expanded CGG repeat-containing cell lines. The increased association of acetylated histones at both histone H3K9 and histone H4 with the *FMR1* locus correlated with CGG repeat length (Figure 4F and Figure S5A, S5B, S5C, S5D). This correlation was best modeled using a pooled analysis of both histone markers and both PCR targets (r^2^ = 0.7337, p = 0.004, Figure 4F). Similarly, the increased association with acetylated histones correlated with FMR1 mRNA expression (Figure 4G, r^2^ = 0.640, p = 0.001; correlations with individual histone markers are shown in Figure S5E, S5F, S5G, S5H). No differences were seen in histone acetylation at the FMR1 locus between confirmed FXTAS cases and premutation cell lines whose clinical information was not known (Figure 4G).

**Figure 5:**
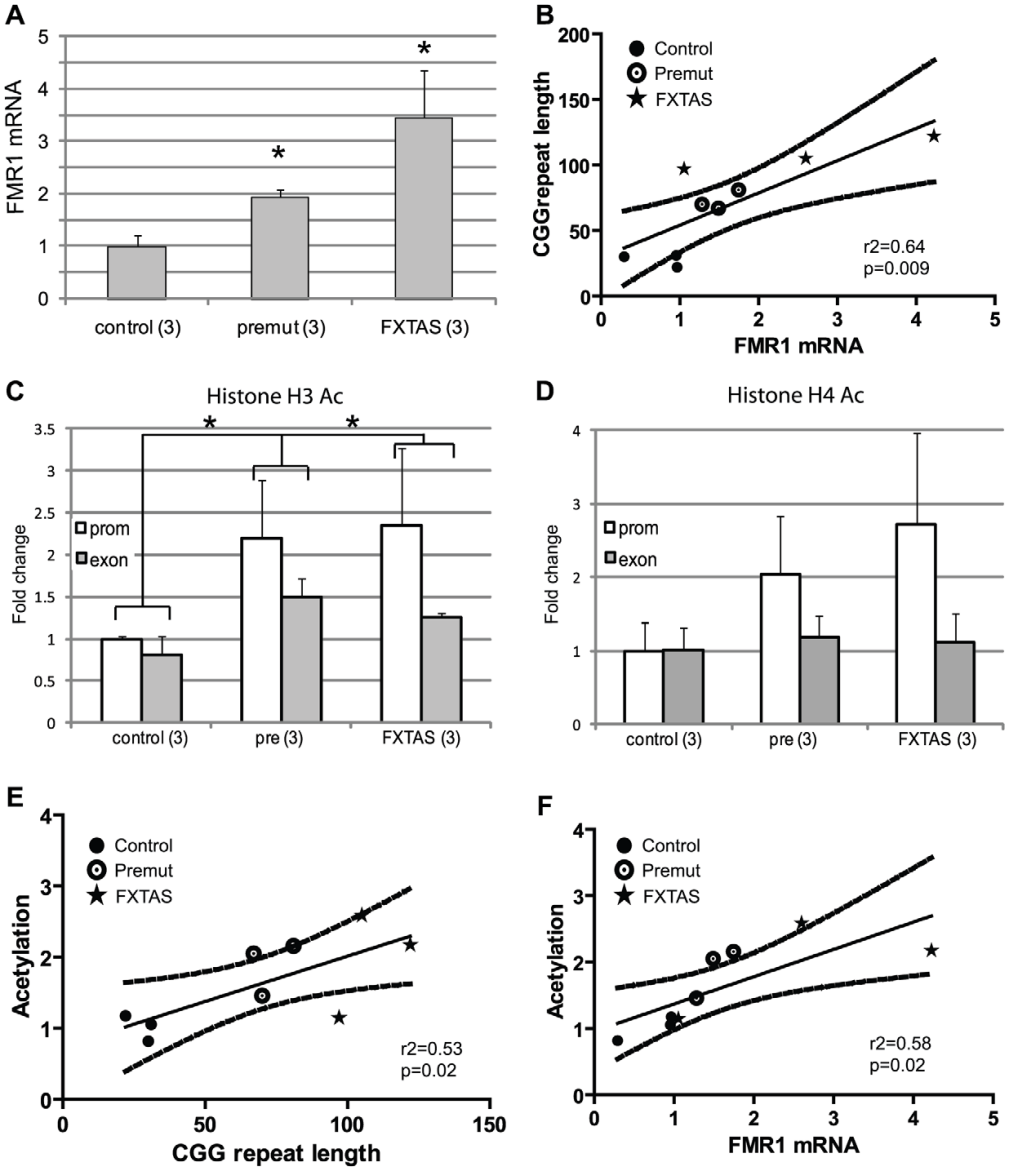
Chromatin alterations at the FMR1 locus in FXTAS patient derived fibroblasts. A) FMR1 mRNA expression in fibroblast cell lines quantified by qRT-PCR, normalized to actin and expressed as fold change from the mean of 3 control cell lines. We used 3 control cell lines (C1(CGG)_31_, C4(CGG)_22_ and C5(CGG)_30_) 3 asymptomatic pre-mutation carrier lines (P3(CGG)_81_, P4(CGG)_70_ and P5(CGG)_67_) and 3 cell lines from FXTAS patients (F1(CGG)_122_, F2(CGG_105_ and F3(CGG)_97_). * = p<0.05 compared to controls. B) Correlation of CGG repeat length and FMR1 mRNA expression. Black dots represent controls, open circles represent asymptomatic pre-mutation carriers, stars represent FXTAS patient-derived cell lines. Straight line reflects best fit and curved lines represent 95% confidence intervals. The correlation is significant (r^2^ = 0.584, p = 0.016). C) ChIP performed on fibroblasts using antibody to Acetylated H3K9. White bars represent primers directed against the *FMR1* promoter, Gray bars against *FMR1* exon 1. Results are normalized to mean value of the control cell lines and expressed as fold change from this cell line. * = p<0.05 of averaged *FMR1* levels from the promoter and exon versus controls. D) ChIP performed on fibroblasts using an antibody directed against Acetylated Histone H4. Pooled analysis grouping of all pre-mutation carrier-derived cell lines was significant for either AcH3K9 alone or a pooled analysis of both (AcH3K9, p = 0.02; AcH4+AcH3K9, p = 0.008), unpaired Students t-test). E) Correlation of CGG repeat number to histone acetylation (r^2^ = 0.44, p = 0.05). F) Correlation of FMR1 mRNA expression and histone acetylation at the *FMR1* locus was significant (r^2^ = 0.582, p = 0.016). Data from individual cell line ChIP experiments are shown in Figure S6.

Although analysis of EBV-transformed lymphoblast cell lines has been a mainstay of research related to chromatin changes associated with Fragile X spectrum disorders, evidence suggests that epigenetic changes in these cell lines do not always reflect the chromatin state in the starting tissues [39]–[41]. We therefore extended our results to patient derived fibroblast samples. Nine previously published and characterized cell lines were obtained as a kind gift from Dr Paul Hagerman: 3 from control patients (repeat ranges from 22–31 CGGs), 3 from asymptomatic pre-mutation carriers (67–81 CGG repeats), and 3 from clinically probable FXTAS patients 97–122 CGG repeats, see materials and methods for details) [42]. As previously described, FMR1 mRNA expression was elevated in both pre-mutation carrier and FXTAS patient-derived cell lines compared to controls, and FMR1 mRNA expression correlated with CGG repeat length (Figure 5A, 5B)[42]. Consistent with our findings in lymphoblastoid cell lines, pre-mutation carrier-derived fibroblasts demonstrated increased association of acetylated histones with the *FMR1* locus surrounding the CGG repeat sequence (Figure 5C, 5D). This association was significant when all pre-mutation carriers were pooled for analysis (p = 0.008 for both AcH3K9 and AcH4 results combined, unpaired Students t-test). Comparisons of individual histone markers were significant only at the AcH3K9 locus, perhaps because of the low number of samples available for analysis. Importantly, the changes in histone acetylation at the *FMR1* locus correlated with FMR1 mRNA expression (Figure 5F, r^2^ = 0.58, p = 0.016) and CGG repeat length (Figure 5E, r^2^ = 0.53, p = 0.02). Individual cell line ChIP results are shown in Figure S6. No significant differences were seen in FMR1 mRNA expression or Histone acetylation between FXTAS derived cell lines and clinically unaffected premutation carriers, although the limited number of cell lines analyzed might preclude detection of a small difference (Figure 5 and Figure S6).

**Figure 6:**
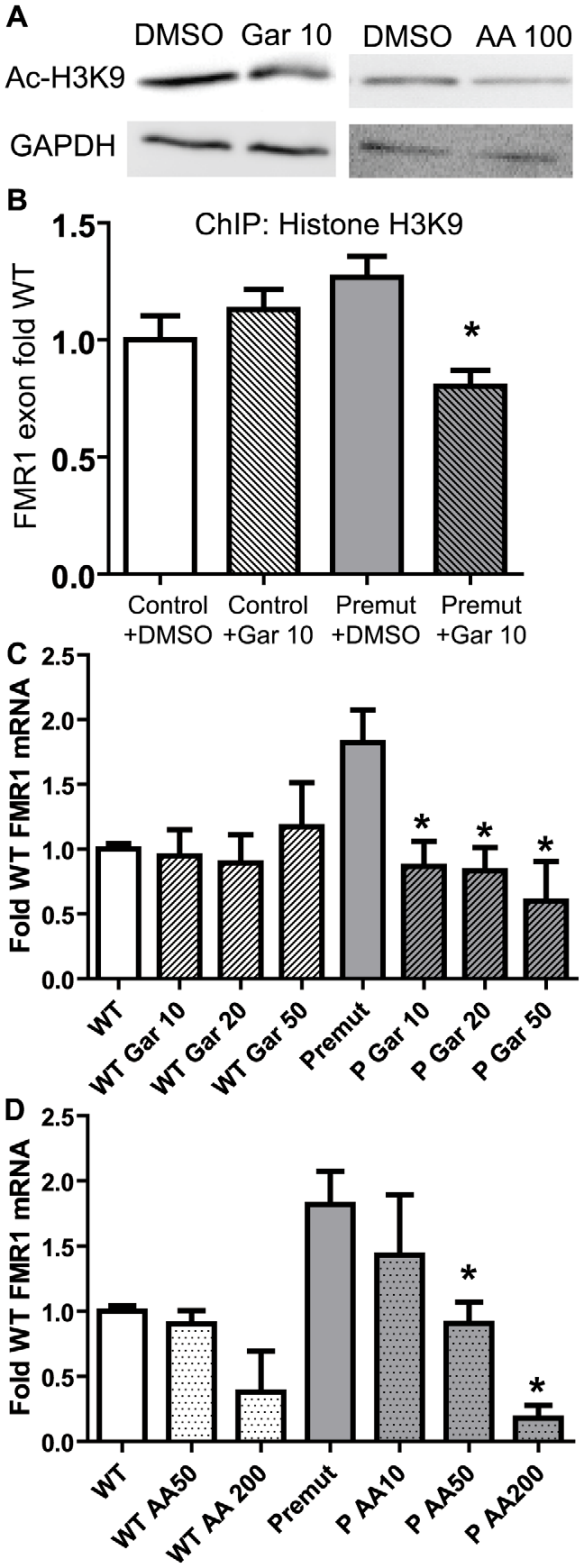
Correction of elevated FMR1 mRNA expression by treatment with histone acetyltransferase inhibitors. A) Pre-mutation carrier derived lymphoblastoid cell lines treated with DMSO, 10 µM garcinol, or 50 µM anacardic acid, for 24 hours are subjected to western blot for Acetyl-Histone H3-K9. GAPDH is shown as a loading control. B) Garcinol decreases chromatin acetylation at the *FMR1* locus selectively in pre-mutation carrier derived cells. Control (C0038.026; (CGG)_30_) or FXTAS patient-derived lymphoblast cells (C014.004; (CGG)_91_) were treated for 24 hours with either 10 µM garcinol or DMSO and subjected to ChIP with acetyl-H3K9 antibodies. Data is expressed as fold change from WT cells treated with DMSO and is a summary of 4 independent experiments. C) Garcinol selectively decreases FMR1 RNA expression in pre-mutation carrier derived cells. The cell lines described in (B) were treated with either DMSO or garcinol at the described doses (in µM) for 24 hours, followed by RNA isolation and qRT-PCR. D) Anacardic acid selectively decreases FMR1 mRNA expression in pre-mutation carrier derived cells. The cell lines described in (B) were treated with either DMSO or Anacardic acid at the specified doses (in µM) for 24 hours and then processed as in (C). For both (C) and (D), FMR1 mRNA levels were normalized to 18S RNA and expressed as fold change from DMSO treated control cells. The data in (C) and (D) are a summary of 3 independent experiments. For all panels, * represents a p<0.05 on paired Students t-test compared to pre-mutation carrier derived cells treated with DMSO.

Unlike some epigenetic modifications, histone acetylation and deacetylation is relatively dynamic[43]. We therefore tested whether we could selectively modify the histone acetylation status of the *FMR1* locus in pre-mutation carrier lymphoblasts. We reasoned that if the homeostatic balance between histone acetyltransferase (HAT) activity and HDAC activity could be shifted by pharmacologically inhibiting HATs, then we might be able to repress the increased FMR1 transcription seen in pre-mutation carrier cell lines. To achieve this we first utilized the broad spectrum HAT inhibitor garcinol[44]. Garcinol treatment of pre-mutation carrier lymphoblasts for 24 hours resulted in modestly reduced global histone acetylation as shown by western blots detecting acetylated histone H3K9 (Figure 6A). To determine whether this drug altered the acetylation status at the *FMR1* locus in FXTAS patient-derived cells, we performed ChIP to acetylated histone H3K9 in cells treated for 24 hours with DMSO or garcinol (10 µM). Garcinol treatment significantly decreased the association of acetylated histone H3K9 with the *FMR1* locus in a probable FXTAS patient-derived cell line (C014.004, (CGG)_91_)to a level similar to that of control cells treated with DMSO (Figure 6B). This dose of drug has no significant effects on histone acetylation at the *FMR1* locus in control cell lines.

FMR1 mRNA is known to have a relatively short half-life of 8 hours in lymphoblasts from both control and FXTAS derived patients[5]. We therefore assessed what effect garcinol treatment had on FMR1 mRNA levels in control and pre-mutation carrier cell lines. Treatment with garcinol for 24 hours at multiple different doses reduced FMR1 mRNA levels by over 50% in pre-mutation CGG repeat cell lines (Figure 6C). Consistent with our ChIP results, there was no effect on FMR1 mRNA levels in control cell lines treated with garcinol.

To confirm these results, we tested the effects of another HAT inhibitor, anacardic acid, on FMR1 mRNA expression. Anacardic acid is a naturally derived compound from cashew nut oil, which has been proposed as a natural anti-cancer drug[45]. As with garcinol, treatment with anacardic acid led to only modest changes in global histone acetylation by western blot (Figure 6A) but a clear, dose-dependent reduction of elevated FMR1 mRNA to near normal levels (Figure 6D). Significantly higher doses of anacardic acid were required to suppress FMR1 mRNA expression in control cell lines, despite the association of acetylated histones with these loci (Figure 4C).

To evaluate whether these HAT inhibitor-induced changes in FMR1 mRNA expression are transient or prolonged, we treated lymphoblasts from one FXTAS patient (CGG91 repeats) with 10 µM garcinol for 24 hours, and then removed the garcinol for 48 hours. Compared to cells treated with DMSO alone for 72 hours, FMR1 mRNA expression in lymphoblasts transiently treated with garcinol had largely returned to normal 48 hours after removal of the HAT inhibitor (Figure S7). In contrast, FMR1 mRNA expression in cells treated for the entire 72 hours with 10 µM garcinol remained depressed (Figure S7). Given the known lower translational efficiency of FMR1 mRNA containing an expanded CGG repeat[7], [22], we also evaluated whether FMRP expression was affected by these drugs. Treatment with 10 µM garcinol for 24 hours had no significant effects on FMRP expression compared to cells treated with DMSO alone (Figure S8D). However, with exposure to 10 µM garcinol for 72 hours, FMRP expression decreased significantly (Figure S8D).

**Figure 7:**
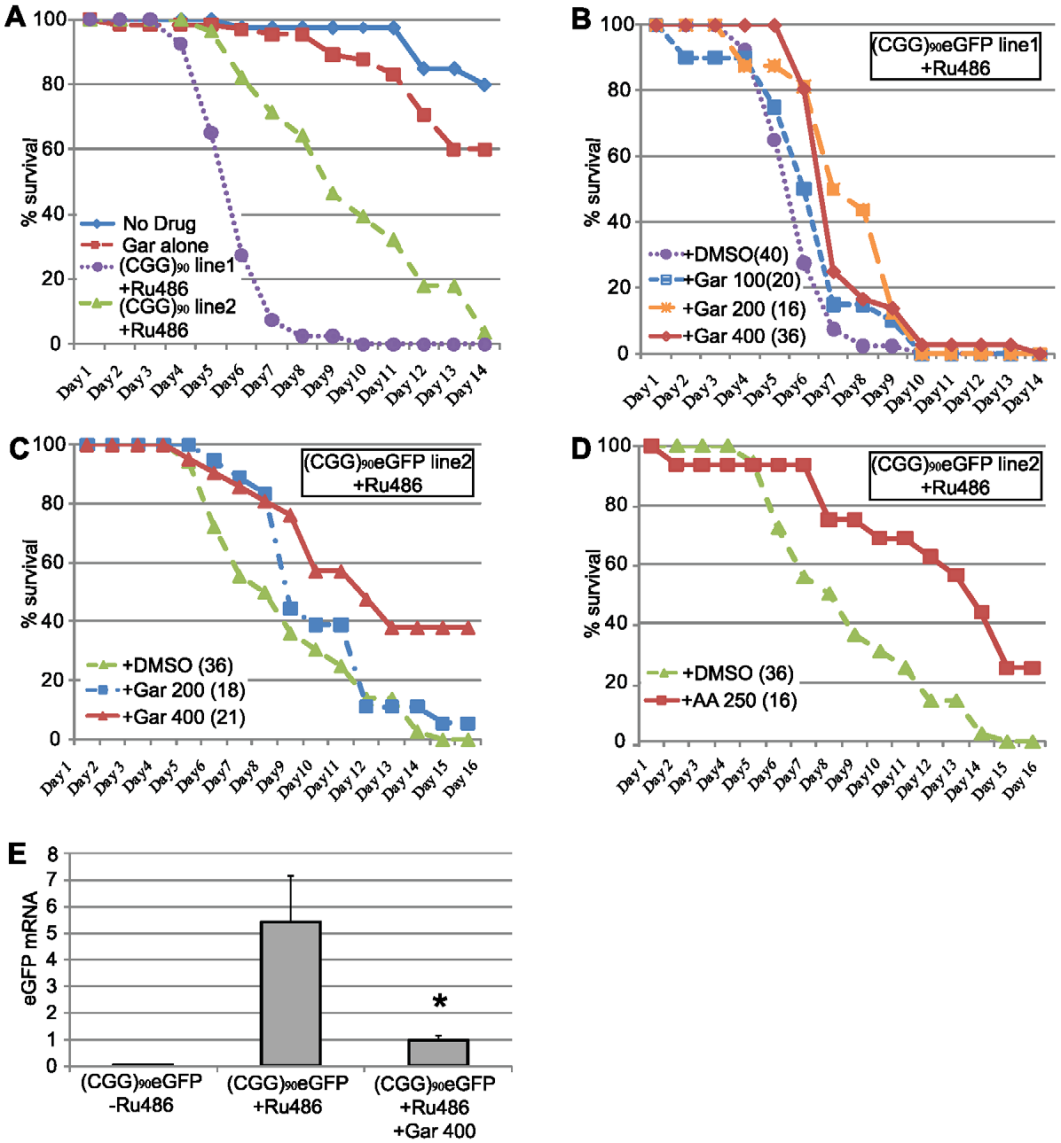
HAT inhibitors extend lifespan in adult (CGG)_90_-eGFP flies. A) young (1–3 days post eclosion) adult (CGG)_90_eGFP line 1or line 2 flies co-expressing a Tubulin Geneswitch driver were transferred to tubes containing normal fly food (blue line), food containing 400 µM Garcinol (red line), or food containing RU486 to activate expression of the ubiquitous driver (Line 1, purple line, Line 2 green line). Data is expressed as % survival over days and represents a summary of two independent experiments. B) For (CGG)_90_eGFP line 1, addition of Garcinol to the food containing RU486 led to a dose dependent increase in lifespan (purple =  RU486+DMSO, blue = RU486+Gar 100 µM, orange = RU486+Gar 200 µM, red = RU486+Gar 400 µM, Log Rank test for trend, p<0.0001). C) Similarly, for (CGG)_90_eGFP line 2, addition of Garcinol to the food containing RU486 led to a dose dependent increase in lifespan, with many flies at the highest dose surviving beyond the end of the study (green =  RU486+DMSO, blue = RU486+Gar 200 µM, red = RU486+Gar 400 µM, Log Rank test (Mantel-Cox), p<0.0001). D) There was also a significant extension of lifespan with addition of Anacardic Acid (250 µM) to the food of (CGG)_90_eGFP line 2 flies (green =  RU486+DMSO, red = RU486+AA 250 µM, Log Rank test (Mantel-Cox), p<0.0001). E) (CGG)_90_eGFP mRNA expression was significantly induced after feeding the flies food containing RU486 for 72 hours. This RU486 activated (CGG)_90_eGFP mRNA expression was suppressed by co-treatment with 400 µM Garcinol (* = paired Students t-test, p<0.01). Data presented are the summary of two independent experiments with n = 10 flies per group for each study. (CGG)_90_eGFP mRNA is quantified in arbitrary units on the y axis.

Because both Garcinol and Anacardic Acid are likely to have broad spectrum effects on transcription, we evaluated the toxicity of these drugs in patient derived lymphoblasts. At low doses of drug (10 µM garcinol, 50 µM anacardic acid) delivered for 24 hours, the time course of most of these experiments, cell viability was unchanged (Figure S8A). As previously published [46], there was cytotoxicity and decreased cell viability evident with higher doses and longer exposure times (Figure S8B). Similarly, attempts to rear *Drosophila* on higher doses of these drugs to test their efficacy *in vivo* demonstrated a dose-dependent reduction in eclosion regardless of genotype, likely due to effects on the chromatin alterations that occur during the transition from larvae to adult flies (Figure S8). However, if *Drosophila* were exposed to these drugs after eclosion, they tolerated addition of significantly higher doses (e.g. 400 µM garcinol) without significant reductions in viability over at least 2 weeks (Figure 7A).

As a proof of principle, we therefore sought to assess whether treatment of adult *Drosophila* with these drugs could affect their lifespan. To accomplish this, we utilized the Geneswitch conditional tissue-specific transgene system. This allows for activation of the uas-(CGG)_90_ eGFP gene ubiquitously after exposure of adult flies to RU-486[47]. In *Drosophila* transgenic for the CGG expansion and co-expressed a ubiquitous GeneSwitch driver (under the Tubulin promoter), there was very few fly deaths over 2 weeks in the absence of RU-486 (Figure 7A, no drug). When exposed to RU-486, however, these flies begin to die 3–4 days post drug for (CGG)_90_eGFP line 1, with 95% of flies dead by day 7 (Figure 7A). For (CGG)_90_eGFP line 2, which has a less severe phenotype when the transgene is expressed in the eye, flies begin to die 5–6 days after exposure and all are dead by day 14 (Figure 7A). Flies expressing the Geneswitch driver alone had no significant alterations in lifespan after exposure to RU-486 (viability = 90% after exposure to RU-486 alone for 14 days, data not shown). The median survival and slope of the survival curves was reliable over multiple trials (the data in Figure 7A represents 3 independent experiments and an n>30 flies/group, log rank test for trend, χ^2^ = 71.48, P<0.0001).

To test whether HAT inhibitors could extend the lifespan of adult uas-(CGG)_90_ eGFP flies triggered to express the transgene ubiquitously, 1–3 day-old adult animals were placed on food that contained either RU-486 +DMSO or RU-486+ increasing doses of Garcinol. As shown in Figure 7B, for (CGG)_90_eGFP line 1, Garcinol led to a dose-dependent increase in survival that was statistically significant at the highest two doses (Garcinol 100 µM versus DMSO, Log Rank test, χ^2^ = 2.92, p = 0.08; Garcinol 200 µM versus DMSO, Log Rank test, χ^2^ = 16.89, p<0.0001; Garcinol 400 µM versus DMSO, χ^2^ = 10.98, p = 0.0003), with a 42% mean increase in lifespan on Garcinol 200 µM. Exposure to Garcinol also significantly extended the lifespan of uas-(CGG)_90_eGFP line 2, with 38% of adult *Drosophila* on 400 µM Garcinol living past the pre-specified endpoint of the trial at 15 days (Figure 7C; Garcinol 100 µM versus DMSO, Log Rank test, χ^2^ = 5.52, p = 0.018; Garcinol 400 µM versus DMSO, χ^2^ = 14.06, p = 0.0002). Similarly, addition of 250 µM Anacardic acid to adult flies also prolonged survival of uas-(CGG)_90_eGFP line 2 flies (Figure 7D; 250 µM AA versus DMSO, Log Rank test, χ^2^ = 20.86, p<0.0001). In congruence with our earlier results showing phenotypic rescue by dHDAC6, these changes in survival were accompanied by a decrease in the expression of the (CGG)_90_eGFP transgene (Figure 7E).

## Discussion

Our results demonstrate that over-expression of three separate histone deacetylases (HDACs 3, 6, or 11) suppress CGG-repeat induced neurodegeneration in a *Drosophila* model of FXTAS. This genetic suppression stems from selective transcriptional repression of the transgene. These findings provided a clue to us that the CGG repeat might induce alterations in local histone acetylation at the FMR1 locus. To evaluate this in the human context, we utilized patient-derived lymphoblasts and fibroblasts from pre-mutation carriers and found that the *FMR1* locus preferentially associates with acetylated histones and this association correlates with elevated FMR1 mRNA expression. Consistent with this finding, histone acetyltransferase (HAT) inhibitors selectively repress the association of acetylated histones with the expanded CGG repeat locus and lower FMR1 mRNA expression to control levels. These same HAT inhibitors are capable of extending the lifespan of (CGG)_90_eGFP fly lines in which the gene is activated ubiquitously in adulthood. These results provide evidence that chromatin alterations associated with the expanded CGG repeat sequence contribute to the elevated FMR1 mRNA levels in pre-mutation expansion carriers. Importantly, the genetic and pharmacologic approaches employed here suggest that these chromatin alterations are modifiable, indicating that the enhanced FMR1 mRNA expression in FXTAS patients could be a viable therapeutic target.

In pre-mutation carriers, FMR1 mRNA levels are elevated 2–10 fold; this finding has been noted in both FXTAS and asymptomatic pre-mutation carrier lymphocytes and fibroblasts, transformed lymphoblastoid cell lines, the brains of expanded CGG-FMR1 knock-in mouse models, and FXTAS patient brains [5], [10], [11], [48]. For repeat expansions in the pre-mutation range, the degree of elevation in mRNA expression generally correlates with the size of the expansion. Several lines of evidence suggest that this elevated mRNA level is due to increased transcription, rather than altered stability, of FMR1 mRNA: 1) There are no changes in RNA stability as measured by the rate of decline of FMR1 mRNA levels after treatment with the transcriptional inhibitor actinomycin[5]; 2) In nuclear run-on assays, mRNA stability does not differ between controls and pre-mutation carriers[25]; and 3) FMR1 mRNA levels are similarly elevated in nuclear and cytosolic fractions of lymphoblastoid cell lines, which would not be expected if mRNA sequestration in nuclear inclusions were a major contributor to increased mRNA accumulation[25].

What triggers this transcriptional activation? There are at least two non-exclusive possibilities. First, because the CGG repeat expansion impairs translation of FMR1 mRNA into protein, the increase could result from a feedback loop driven by lower levels of the Fragile X Mental Retardation Protein, FMRP. Alternatively, the increase in transcription could result from intrinsic changes in local chromatin structure induced by the expanded CGG/CCG repeat itself [49]. In support of this latter hypothesis, pre-mutation expanded non-methylated CGG/CCG tracts exclude nucleosomes *in vitro* and preferentially associate with acetylated rather than non-acetylated histones, which would be predicted to enhance basal transcriptional activity [26], [27]. Moreover, recent genome-wide evidence suggests that non-methylated CpG islands are predominantly associated with markers of active chromatin, including acetylated histones[50]. Consistent with a primary effect of the CGG expansion in *cis* on transcription, we observed increased (CGG)_90_-eGFP mRNA expression in two *Drosophila* lines compared to control eGFP mRNA expression with the same promoter and driver (Figure 3A). Although these findings could be artifacts explained by insertion site variation, we suggest instead that they may reflect intrinsic effects of the CGG repeat on DNA structure that influence transcription efficiency.

Extensive work has characterized the regulation of basal and activity-dependent transcription of the *FMR1* gene with a normal sized CGG repeat versus the fully expanded repeat of Fragile X Mental Retardation (FXS). In most FXS patients both an upstream CpG island and the expanded CGG/CCG repeat are hypermethylated[51], [52]. This hypermethylation drives the chromatin state at this locus from euchromatin to heterochromatin, and the H3 and H4 histones associated with the *FMR1* promoter and 5′UTR become hypoacetylated [36]–[38]. This combination of hypermethylation and heterochromatin results in transcriptional silencing of the *FMR1* gene and the symptoms of FXS. Treatment of FXS patient lymphoblasts with general histone deacetylase (HDAC) inhibitors partially reverses transcriptional silencing in lymphoblastoid cell lines[53] and treatment of FXS patients with valproic acid, an antiepileptic and mood stabilizing drug with HDAC inhibitor activity, has produced promising preliminary results [54]. However, full reactivation of the gene also usually requires demethylation of the DNA. Previous work has also characterized chromatin changes in fibroblasts and lymphoblasts derived from unmethylated full mutation(>200 CGG repeats) patients [22], [23], [55]. In these cases, FMR1 mRNA expression is close to normal but protein expression remains very low or absent because of translational inefficiency. In unmethylated full mutation patient-derived cells, histone H3/H4 acetylation remained significantly depressed compared to controls and remains closer to methylated full mutation carrier levels despite close to normal FMR1 transcription[22], [23], [55]. However, histone methylation and demethylation events are more predictive of transcriptional activity in these cell lines.

Our results concur with these previously published studies and provide new information about the chromatin state in FXTAS patient-derived cells. In lymphoblasts and fibroblasts with an expanded non-methylated pre-mutation length CGG repeat, we observe an increase in acetylated H3 and H4 histones at the *FMR1* locus surrounding the CGG repeat. In light of previously published data, our results suggest that the modestly expanded FXTAS repeat sequence alters chromatin structure in *cis*, largely through preferential association with acetylated histones, which in turn promote transcription at the *FMR1* locus, driving up FMR1 mRNA levels and potentially exacerbating RNA mediated toxicity. It remains unclear, however, why a pre-mutation repeat expansion behaves differently than an unmethylated full mutation length repeat expansion[22]. Further, we have not yet analyzed other potential chromatin changes at the *FMR1* locus such as histone methylation, which might contribute to transcriptional regulation in pre-mutation carriers. Future studies will be needed to address these questions.

Our findings do not exclude an independent contribution to elevated FMR1 transcription from transcription factor activation in response to inefficient FMRP translation. In both pre-mutation carriers and full expansion unmethylated Fragile X cases, the CGG repeat interferes with translation by altering the loading of the FMR1 mRNA onto polysomes[7], [8], [22], [24] Several lines of evidence, however, suggest that transcription factor activation is not the dominant cause of elevated FMR1 levels. Perhaps most convincing are data from a fragile X syndrome patient with a point mutation that encodes a nonfunctioning but stable FMRP. In this patient, who has a normal CGG repeat, FMR1 mRNA levels are normal [56]. Similarly, a knock-in model of this same mutation into the *FMR1* locus in mice recapitulates many features of the Fragile X phenotype but FMR1 mRNA levels were not altered[21]. In addition, FMR1 levels are elevated in most tissues in FXTAS patients and CGG repeat expressing mice, despite probable compensatory mechanisms related to FXR1 and FXR2 functions in these tissues and the lack of a significant tissue phenotype in FXS patients [3], [10], [11], [57]; it is unclear why there would be a strong feedback trigger for transcription of FMR1 in tissues where its function is not clearly essential. However, the role of decreased FMRP translational inefficiency in FMR1 pre-mutation transcription will need to be addressed empirically in future studies.

Pre-mutation carriers demonstrate a reduced penetrance for FXTAS. It is estimated that only 40% of pre-mutation carrier males will manifest symptoms prior to death[1], [58], but what features drive the penetrance of FXTAS remain unknown. Epigenetic changes are one possible mechanism. This study was not designed or powered to assess differences between asymptomatic pre-mutation carriers and FXTAS patients. However, we did not observe any significant differences in FMR1 mRNA expression or histone acetylation patterns of asymptomatic pre-mutation carriers versus FXTAS patient derived fibroblasts. What little differences were seen are largely attributable to larger CGG repeat expansions in the FXTAS patient derived cell lines. Larger studies utilizing pathology confirmed FXTAS cases and controls will be required to fully answer this question. Evaluating what factors influence the incomplete penetrance in this disorder, including further analysis of the role played by the antisense message that is transcribed through the CGG/CCG repeat [59], [60], is an area ripe for further research. In addition, future work will be needed to more critically address how this CGG/CCG repeat can influence transcription. For example, is the presence of the repeat alone adequate to induce alterations in histone acetylation and transcriptional activation or is the surrounding FMR1 5′UTR important in the ability of the CGG repeat to influence transcription? Does the CGG repeat have to be transcribed to cause these alterations? And what role does antisense CCG repeat transcription play in both transcriptional and post-transcriptional regulation of FMR1?

HDAC inhibition has emerged as a potential therapeutic strategy in numerous neurodegenerative diseases, including polyglutamine disorders [31], [34]. In contrast, our work identifies three different classes of HDACs as potential *suppressors* of CGG repeat induced neurodegeneration. Consistent with this finding, treatment of patient lymphoblasts with Histone Acetyltransferase (HAT) inhibitors suppressed FMR1 mRNA expression selectively in pre-mutation carrier derived cells. A few caveats need to be addressed to fully understand these findings. Although the tested HDACs likely rescue the phenotype by suppressing CGG repeat-dependent transgene transcription, some of their activity could reflect other effects, including altering transcription of other genes critical to CGG repeat-dependent toxicity. HDAC6 in particular has cytoplasmic functions linked to autophagy that could mitigate the CGG repeat neurodegenerative phenotype. A major role for autophagy in the suppression we observed is unlikely, however, given that siRNA directed against a critical component of the autophagy pathway has no effect on CGG repeat-dependent phenotypic rescue by dHDAC6 (Figure 1). In contrast, this same siRNA prevents dHDAC6-dependent rescue of a polyglutamine phenotype ([30] and data not shown). It is worth noting that dHDAC6 did not suppress transcription of eGFP alone at two different insertion sites or of a polyglutamine disease gene (androgen receptor with 52 or 120 CAG repeats) in *Drosophila* [30], suggesting that the HDAC-mediated transcriptional repression we observe may be a CGG repeat-specific event.

We demonstrate that two different histone acetyltransferase inhibitors, anacardic acid and garcinol, can lower FMR1 mRNA levels in pre-mutation carrier derived lymphoblasts. For garcinol, this suppression of FMR1 mRNA expression correlates with decreased association of acetylated histones with the *FMR1* locus. Intriguingly, the effects of these drugs, at least at lower doses, seem to be somewhat selective for the expanded CGG repeat effects in the *FMR1* locus, as the effects on chromatin structure and FMR1 mRNA expression are less pronounced in control cell lines and the global acetylation state of histones in the cells are not dramatically altered. These data support a model whereby the histone acetylation state and thus the chromatin structure at the *FMR1* locus is more dynamic in the setting of an expanded CGG repeat. As with the effects of HDAC overexpression in the *Drosophila* model, the effects of HAT inhibitors on FMR1 levels in lymphoblasts may be pleotropic. Future work should better delineate how these drugs achieve a reduction of FMR1 levels.

Regarding the potential use of HAT inhibitors as therapeutic agents, both garcinol and anacardic acid are naturally occurring chemicals that have been used in humans as homeopathic agents. In addition, both have been proposed as possible anti-cancer agents clinically [61], [62]. However, these drugs lead to a global repression of transcription and cytotoxicity in dividing cells at doses necessary to achieve significant histone acetyltransferase inhibition (see Figure S8 and [44], [61], [63], [64]). Thus, they are likely to be too toxic with significant non-specific off target effects to achieve transcriptional repression of a single gene in patients. Consistent with this, we were unable to suppress CGG-repeat induced neurodegeneration in our *Drosophila* model with HAT inhibitors delivered prior to eclosion due to significant non-specific toxicity (Figure S8 and data not shown). Even if *FMR1* transcription can be efficiently and selectively targeted, a balance will need to be achieved between lowering FMR1 mRNA to a non-toxic level while maintaining FMRP expression, given the known phenotype associated with the loss of FMRP (Fragile X mental retardation). Consistent with this concern, we see a significant reduction in FMRP levels in pre-mutation-carrier lymphoblasts after treatment with Garcinol for 72 hours (Figure S8D). Thus, our findings to date are best viewed as a proof of principal that FMR1 transcription in pre-mutation carrier cells are amenable to pharmacologic and genetic manipulation, rather than indicative that these agents should serve as a basis for therapeutic development. Our results also raise the potential concern that drugs that display activity as HDAC inhibitors might be harmful in FXTAS patients. For example, Valproic acid, a drug that may be considered for patients with neuropsychiatric symptoms or seizures, is known to have HDAC inhibitor activity. This theoretical risk may warrant investigation in FXTAS patients.

In summary, we provide evidence that HDAC expression *in vivo* or pharmacologic treatment with HAT inhibitors in patient cells can correct the transcriptional upregulation associated with pre-mutation length expanded CGG repeat sequences. Our data support a model whereby the expanded CGG repeat sequence in the *FMR1* gene in FXTAS patients alters local chromatin structure in *cis* to favor increased FMR1 transcription. It remains unknown how the expanded repeat drives such changes in chromatin, but *in vitro* data suggest it may stem from changes in secondary DNA structure that discourage association with de-acetylated histones and nucleosomes[26]. These changes in DNA structure then drive FMR1 overexpression in FXTAS and contribute to neurodegeneration. Importantly, our results suggest that these alterations in chromatin structure at the *FMR1* locus are dynamic and modifiable, such that various genetic and pharmacologic manipulations of the state of histone acetylation at and near the CGG repeat can change the level of transcription in human cells and in animal models. Our findings underscore the importance of developing more specific modifiers of FMR1 transcription with the long term goal of developing preventive therapy for FXTAS patients.

## Materials and Methods

### Fly stocks

All *Drosophila experiments* were performed on standard food in 25°C incubators unless otherwise noted. Uas-(CGG)_90_eGFP lines 1 and 2 (designated lines BD and BC, respectively) were a kind gift from Peng Jin at the University of Emory and have been previously described [13], [18], [19]. Stability of the CGG repeat was confirmed by PCR and sequencing using C and F primers as described previously [13], [65]–[67]. Uas-dHDAC6L #13 and UAS-dHDAC6kd flies have been previously described and characterized [30]. UAS-HDAC11kd #1B3 and UAS-dHDAC6KD were obtained as a kind gift from Dan Garza at Novartis Pharmaceuticals. Tub-Gal4, Gmr-GAL4, Uas-eGFP and UAS-LacZ fly lines were obtained from the Bloomington *Drosophila* Stock Center (Bloomington, IN).

The cDNA for *Drosophila* HDAC3 and HDAC6 were amplified from the expressed sequence tag (EST) clones LD23745 and LD43531, respectively. The coding region for *Drosophila* HDAC11 was generated by positional cloning from EST clone CG31119-RA. Primers were created to insert 5′ Kpn I and 3′ Xba I restriction sites and the constructs were ligated into the pAc5.1/V5 vector (Invitrogen, Carlsbad, CA) as previously described [28]. Primers were then generated to introduce Spe I at the 5′ end just upstream of the Kpn I site and Avr II at the 3′ end of the dHDAC constructs maintaining the V5 tag, 6xHIS, stop codon and the SV40 pA sites from the pAc5.1/V5 vector and was subcloned into a pINDY6 vector [68] cis to the yeast upstream activating sequence (UAS). The pINDY6 vector containing the dHDACs was used to generate transgenic fly lines using standard germ-line transformation by Genetic Services (Cambridge, MA). The dHDAC expression was activated in genetic crosses trans to the yeast GAL4 transcription factor, which was in turn regulated by the eye-specific promoter GMR upstream of the yeast GAL4 cDNA.

### Eye images

Eye phenotypes of 1–2 day-old anesthetized flies were evaluated with a Leica MZ APO stereomicroscope and photographed with a Leica DFC320 digital camera as previously described [30]. For each genotype and condition, at least 100 flies were evaluated. For fluorescent images of *Drosophila* heads to evaluate eGFP expression, animals were anesthetized with CO2, decapitated, and immediately imaged on a glass coverslip by epifluorescence on an inverted Olympus IX71 microscope. All images were taken at the same exposure. Images were processed using Slidebook 4.0 software and subtracted for background auto-fluorescence based on images of non-transgenic flies. Images are 10x in coronal orientation. For quantitation of the severity of the eye phenotypes, a degeneration scale was used as previously described [30]. Briefly, flies were given a score between 0 and 10 on the following scale: 1 point for extranumerary bristles, 1 point for abnormal bristle orientation, 1 point for oomatidial fusion, 1 point for oomatidial pitting, 1 point for retinal collapse. In addition, 1 point was added if more than 5% of the eye was affected, 4 points was added if more than 50% of the eye was affected. For quantitation, at least 20 flies per group were utilized.

### SEM

SEM samples were processed as previously described [30]. Briefly, flies were collected and fixed in 2.5% glutaraldehyde (EMS) in PBS and post-fixed for 15–30 minutes in 1.5% osmium tetroxide (Stevens Metallurgical) in PBS. Samples were then dehydrated in ethanol, immersed in hexamethyldisilazane (Polysciences Inc.) and dried in a desiccator for three days. Specimens were then coated with gold:palladium using a Denton DV-503 vacuum evaporator, and analyzed using an AMRAY 1820D scanning electron microscope.

### RT-PCR

Semi-quantitative RT-PCR analysis in cells and *Drosophila* was performed as previously described [30], [69]. Briefly, flies of the specified genotypes were decapitated under anesthesia and the heads were flash frozen on dry ice. After crushing the heads with a pipette tip, total RNA was extracted using Trizol reagent (Invitrogen, San Diego, CA) according to manufacturer's protocols. Lymphoblastoid Cell lines were homogenized directly in Trizol prior to extraction. Following spectrophotometric quantification, 1 µg of total RNA was reverse-transcribed (iScript cDNA Synthesis Kit; BioRad, Hercules, CA) and the resulting cDNA subjected to quantitative real-time PCR analysis with gene specific primers [38] as follows: eGFP: (Fwd: CTGCTGCCCGACAACCA; Rev: GAACTCCAGCAGGACCATGTG), FMR1: (Fwd: CCGAACAGATAATCGTCCACG; Rev: ACGCTGTCTGGCTTTTCCTTC), 18S Fly: (Fwd: CGGCTACCACATCCAAGGAA; Rev: GCTGGAATTACCGCGGCT), 18SHum: (Fwd:CAGCCACCCGAGATTGAGCA; Rev:TAGTAGCGACGGGCGGTGTG), β ~Actin: (Fwd: GGCATCCTCACCCTGAACTA; Rev:AGAGGCGTACAGGGATAGCA), GAPDH: (Fwd: AGAAGGCTGGGGCTCATTTG Rev: AGGGGCCATCCACAGTCTTC).

PCR analysis was performed using the iQ SYBR Green Supermix in a myiQ Single Color RTPCR system (BioRad). All runs included a standard dilution curve representing at least 10x and 0.01X the RNA concentration utilized for all primer sets to insure linearity. Further, equivalent efficiency of individual primer sets was confirmed prior to data analysis. For *Drosophila*, the levels of (CGG)_90_-eGFP mRNA were normalized to those of 18S RNA or GAPDH mRNA for each sample run and expressed as a ratio of levels found UAS-eGFP lines (fold control expression). For lymphoblast derived mRNA quantitation, levels of FMR1 mRNA were normalized to actin RNA or 18S RNA and expressed as a ratio of mean expression of normal repeat length cell lines (fold control expression). All samples were run in triplicate and all data represent at least three independent experiments.

### Lymphoblastoid cell lines

Three cell lines were obtained as a kind gift from Stephanie Sherman at Emory University. Their brief clinical history is below:

1. C0140.004 (CGG 91), Definite FXTAS; 62 y.o. man with onset of symptoms around age 50, Middle cerebellar Peduncle (MCP) sign on MRI, postural kinetic tremor early, developed gait ataxia, cerebellar tremor, voice tremor and head titubation; no nystagmus or parkinsonian features.
2. C0051.004, (CGG 90) Definite FXTAS; Onset at 59, postural tremor with mild gait imbalance. MCP sign on MRI;
3. C0038.026, asymptomatic male with 30 CGG repeats.

All other lymphoblastoid cell lines were obtained from the Coriell Cell Repository (Coriell, Camden, NJ).

1. GM09237, symptomatic FXS male with >900 CGG repeats.
2. GM06891, 29YO male with 118 CGG repeats, clinical status for FXTAS unknown.
3. GM06892, 84YO male with 93 CGG repeats, clinical status for FXTAS unknown.
4. GM06895, 55YO male with 23 CGG repeats; related to 6891 and 6892, clinical status unknown.
5. GM20244, 43 YO male with 41 CGG repeats, clinical status unknown.

Repeat size was confirmed by PCR for all lines except for the FXS patient line, as previously described using C and F primers [65], [67]. Cells were grown in RPMI 1640 media with Glutamine pre-added (Invitrogen) and 12% fetal bovine serum plus antibiotics as previously described[12].

### Fibroblast cell lines

Nine fibroblast cell lines were obtained as a kind gift from Dr Paul Hagerman and colleagues (University of California at Davis). The clinical state of these patients has been described previously and all cell lines were obtained from males [42]. Nomenclature used follows that used in the publication where these lines were first published [42]. Briefly, there are three control lines: C1 (CGG31), C4 (CGG22) and C5 (CGG30). There are three cell lines from asymptomatic pre-mutation carriers (all at least 68 years old): P3 (CGG81), P4 (CGG70) and P5 (CGG67). Lastly, there are three cell lines from patients with clinically definite FXTAS based on established criteria[70]: F1 (CGG122), F2 (CGG105) and F3 (CGG97). Fibroblasts were cultured and maintained as previously described[42] and repeat size was confirmed by PCR for all lines using C and F primers [65], [67].

### Chromatin immunoprecipitation

Formaldehyde Cross linking ChIP was performed according to previously published commercial protocols using a ChIP kit (Millipore, Temecula, CA) [71]. Antibodies to Histone H3 acetyl K9 (ab10812, Abcam, Cambridge, MA), Acetyl Histone H4 (abCS200571, Millipore), or rabbit IgG (Millipore) were used to immunoprecipitate the DNA/protein complexes overnight. After reversal of crosslinks and DNA isolation, real time PCR was conducted on equal concentrations of input and IP derived DNA in triplicate. Primers used against the *FMR1* promoter and exon1 were previously published [38]. Control primers to human GAPDH optimized for ChIP were obtained commercially (Millipore). FMR1 DNA quantity from IP was normalized to input FMR1 DNA. This number was then re-normalized to the mean of the control cell lines and expressed as fold change from this mean. For both selective antibodies, IP qPCR was similar to input and >10x of ChIP with rabbit serum or non-specific antibodies (e.g. Beta-gal) for both FMR1 and GAPDH primers (Figure S3). PCR primers targeted within 100 bp upstream or downstream of the CGG repeat showed similar results, but with altered efficiency due to high GC content, complicating quantification (data not shown).

### Drug treatment of cell lines

Anacardic Acid was obtained from Calbiochem and Garcinol was obtained from Sigma. All drugs were dissolved in DMSO. Cells were treated for 24 or 72 hours (Garcinol 10, 20, or 50 µM or Anacardic Acid at 10, 50, 200, or 500 µM) with drug added directly to the culture media. Both 18S RNA and actin were used as internal controls for qRT-PCR experiments. FMR1 mRNA expression was normalized to 18S RNA in all drug experiments rather than actin because at the highest doses of both drugs, there were alterations in actin, GAPDH and c-myc mRNA levels (data not shown).

### Cell viability and cytotoxicity assays

Determination of cell viability and cytotoxicity was performed using a MultiTox-Glo Multiplex Cytoxicity Assay kit from Promega (Madison, WI, USA) according to the manufacturers' protocol. Additional viability assays were done using trypan blue exclusion with similar results.

### Lifespan studies in *Drosophila*

RU-486(Sigma) or Vehicle(Ethanol) was added to standard fly food at a final concentration of 200 µM as previously described [72]. Uas-(CGG)_90_eGFP line 1 and line 2 fly lines were crossed with flies expressing a Gene-Switch Tubulin-Gal4 driver (Tub5, a kind gift from Scott Pletcher at the University of Michigan). At 0-3 days post eclosion, flies of the specified genotype were switched to food containing drug or vehicle at 25C at 60% humidity. Viability was assessed daily for 15 days or until all flies were dead, whichever came first. This latter time point was chosen because of concerns over the stability of RU-486 and HAT inhibitors over time.

For drug studies, Garcinol (from a stock solution of 100 mM), Anacardic acid (stock solution of 250 mM) at various concentrations or an equal volume of vehicle (DMSO) were added to food containing RU-486 as described above. Different volumes of DMSO had the same viability as RU-486 alone, so these results were pooled for statistical analysis. Data in Figure 7 represents a summary of at least 3 independent experiments with all results pooled.

For viability assays done pre-eclosion, uas-eGFP/CyO (Chr 2) or uas-(CGG)_90_eGFP/CyO line 1 and line 2 fly lines (Chr 2) were crossed with flies expressing a tubulin-Gal4 driver (Chr 3). Viability was assessed by determining the ratio of progeny carrying the balancer chromosome (CyO) to those carrying the UAS-transgene. Data are representative of at least 4 experiments.

### Western blotting

Western blotting to FMRP was performed as previously described [73]. Briefly, cell lysates were lysed in RIPA buffer, sonicated, and quantified by the method of Bradford. Equal protein amounts were then boiled in 2x Laemmeli buffer and run on a 12% SDS polyacrylamide gel. After transfer to PVDF membrane at 36 amps for 1.5 hours at 4C, the blots were incubated with antibodies to FMRP (Millipore 1∶1000 O/N at 4C) and GAPDH (Santa Cruz Biotechnology, used at 1∶1000 O/N at 4C). Images from westerns are representative of at least 2 independent experiments with similar results.

### Statistical analysis

For all graphs, error bars represent Standard error of the mean calculated in Microsoft Excel. For comparisons of single groups, a Student's two sided T-Test was performed. For drug studies in lymphoblast cell lines, these were done as a paired T-Test. For all others, unpaired T-tests were performed.

Other statistical analyses were performed using GraphPad Prism. For correlation analysis, data are presented as the best fit curve with 95% confidence intervals. r^2^ (coefficient of determination) is included as a measurement of variance explained by the selected variable. An F test was performed to determine statistical significance. For comparisons to CGG repeat length, a Spearman correlation was used to account for the non-parametric distribution of repeat lengths in our samples. All other comparisons utilized a Pearson correlation. For survival analysis, a Log-rank (Mantel-Cox) test or a Log-rank test for Trend was performed. Flies surviving past the specified end of the study (15 days) were assigned a survival time of 15 days for statistical purposes.

## Supporting Information

Figure S1 The HDAC inhibitor SAHA exacerbates CGG repeat dependent neurodegeneration. Scanning electron microscopy (A–D) or light microscopy (E–G) images were obtained from flies expressing eGFP (A) or (CGG)_90_-eGFP line I (B–G). Flies were reared at 25C in standard fly food containing either DMSO (B,E) or the broad spectrum HDAC inhibitor SAHA at 150 µM (C, F) or 300 µM (A,D,G). Images are representative of greater than 25 flies per treatment group.
(3.86 MB TIF)

Figure S2 The HDAC inhibitor SAHA partially suppresses rescue by dHDAC6 of CGG repeat dependent neurodegeneration. Representative SEM images from *drosophila* co-expressing (CGG)_90_-eGFP line I and UAS- dHDAC6 reared on DMSO(A), or SAHA at 150 µM (B) or 300 µM (C). Quantitation of multiple flies reveals a significant worsening of the phenotype in flies reared on SAHA. (* = p<0.01 compared to (CGG)_90_-eGFP line I alone, + = p<0.01 compared to (CGG)_90_-eGFP line I x dHDAC6 by a Students unpaired t-test).
(1.38 MB TIF)

Figure S3 Chromatin immunoprecipitation from pre-mutation carrier cells. Input is crosslink reversed DNA not subjected to immunoprecipitation. Data are expressed as a ratio to the *FMR1* exon 1 signal from the input material. There is enrichment of the *FMR1* exon 1 locus in pre-mutation carrier lymphoblast cell lines when ChIP is performed against either Ac-histone H3K9 or Ac-histone H4 compared to IgG alone. * = P<0.001 by unpaired t-test compared to IgG immunoprecipitation alone.
(0.05 MB TIF)

Figure S4 Chromatin immunoprecipitation results from each lymphoblast cell line. ChIP was performed 3–4 times on each of 13 different lymphoblastoid cell lines. The data from each cell line is provided. FMR1 mRNA and ChIP results for all samples were normalized to cell line GM20244 (CGG)_41_ during each qPCR run to allow for run to run comparisons. Cell lines are divided into normal repeat lengths (A–G), Clinically probable FXTAS patient-derived cell lines (H,I), and pre-mutation carrier derived cell lines whose clinical status is unknown (J–M).
(0.50 MB TIF)

Figure S5 Correlations between ChIP AcH3K9 and AcH4 to CGG repeat number and FMR1 mRNA expression. For each graph, solid black dots =  control cell lines, stars =  confirmed FXTAS cases, open circles =  pre-mutation carriers whose clinical status is unknown. The central line is the linear best fit. Curved dashed lines are 95% confidence intervals. The r^2^ and significance for each correlation is shown in each graph. ChIP to Ac H3K9 correlated with CGG repeat number using PCR primers directed at either the FMR1 promoter (Fig A, FMR1 prom AcH3K9 to CGG#) or the FMR1 exon (Fig B, FMR1 exon AcH3K9 to CGG#). Correlation of ChIP to Ac H3K9 and FMR1 mRNA expression was significant using PCR primers directed at the FMR1 exon (Fig F, FMR1 exon H3K9 to FMR1 mRNA), but not the FMR1 promoter (Fig E, FMR1 prom AcH3K9 to FMR1 mRNA). ChIP against Ac H4 correlated with CGG repeat number (Fig C,FMR1 prom AcH4 to CGG#; Fig D, FMR1 exon H4 to CGG#) and FMR1 mRNA expression (Fig G, FMR1 prom AcH4 to FMR1 mRNA; Fig H, FMR1 exon AcH4 to FMR1 mRNA) using PCR primers directed at either the FMR1 promoter or the FMR1 first exon.
(0.67 MB TIF)

Figure S6 ChIP and FMR1 mRNA results from individual fibroblast cell lines. A) ChIP against Ac H3K9 or pan acetylated H4 and FMR1 mRNA expression normalized to Actin mRNA expression is shown for each sell line. All data is presented as fold change from Control fibroblast line #C1. Error bars represent SD from 2–3 independent experiments. B–E) Correlations of individual acetylated chromatin marks as determined by ChIP (y-axis) with CGG repeat number (x-axis). F–I) Correlation between individual Acetylated Chromatin marks as determined by ChIP (y-axis) with FMR1 mRNA expression (x-axis). For each, r^2^ and significance of Pearson correlation is provided as an inset.
(0.44 MB TIF)

Figure S7 Garcinol effects on FMR1 expression are transient. Lymphoblasts derived from a patient with probable FXTAS (#C0014.004, CGG91 repeats) or from a control patient were treated for 24 or 72 hours with 10 µM garcinol or DMSO. After 24 hours, some Garcinol treated cells had their media changed to include only DMSO for 48 hours. Equal numbers of cells were harvested and mRNA was extracted and quantified by qPCR. FMR1 mRNA levels are normalized to 18S mRNA and expressed (approximately) as a ratio to FMR1 expression in DMSO treated cells. There is a significant reduction in FMR1 mRNA expression in FXTAS cells treated for 72 hours with Garcinol, but there is no significant difference in FMR1 expression in cells treated with Garcinol for only 24 hours and then switched to vehicle. *P = 0.05, Students t-test versus DMSO treated cells.
(0.12 MB TIF)

Figure S8 Toxic effects of HAT inhibitors on lymphoblast cells and fly eclosion. Exposure to HAT inhibitors has been reported as toxic to cancer cells. We therefore assessed the effects on viability and cytotoxicity of various doses of HAT inhibitors on lymphoblast cell lines. A) Treatment for 24 hours with garcinol (10 µM) or anacardic acid (50 µM) at the minimally effective dose for altering FMR1 mRNA expression did not alter cell viability. B) However, at higher doses (20–50 µM garcinol) and with longer exposures (48–96 hrs), these drugs were toxic to lymphoblast cell lines. C) These drugs also blocked eclosion of flies reared on doses greater than 25 µM. Garcinol or 75 µM anacardic acid, which precluded performing some phenotypic rescue experiments. D) Consistent with the decrease seen in FMR1 mRNA expression, FMRP levels are stable after 24 hours of exposure to10 µM garcinol but are significantly depressed after 72 hours of exposure, although interpretation of this later time point may be complicated by decreased cell viability (note lower molecular weight degradation products of FMRP at this time point).
(0.30 MB TIF)

## References

[1] Jacquemont S, Hagerman RJ, Leehey MA, Hall DA, Levine RA (2004). Penetrance of the fragile X-associated tremor/ataxia syndrome in a premutation carrier population.. JAMA.

[2] Hagerman PJ, Hagerman RJ (2004). The fragile-X premutation: a maturing perspective.. Am J Hum Genet.

[3] Greco CM, Berman RF, Martin RM, Tassone F, Schwartz PH (2006). Neuropathology of fragile X-associated tremor/ataxia syndrome (FXTAS).. Brain.

[4] Greco CM, Hagerman RJ, Tassone F, Chudley AE, Del Bigio MR (2002). Neuronal intranuclear inclusions in a new cerebellar tremor/ataxia syndrome among fragile X carriers.. Brain.

[5] Tassone F, Hagerman RJ, Taylor AK, Gane LW, Godfrey TE (2000). Elevated levels of FMR1 mRNA in carrier males: a new mechanism of involvement in the fragile-X syndrome.. Am J Hum Genet.

[6] Tassone F, Hagerman RJ, Taylor AK, Mills JB, Harris SW (2000). Clinical involvement and protein expression in individuals with the FMR1 premutation.. Am J Med Genet.

[7] Primerano B, Tassone F, Hagerman RJ, Hagerman P, Amaldi F (2002). Reduced FMR1 mRNA translation efficiency in fragile X patients with premutations.. RNA.

[8] Chen LS, Tassone F, Sahota P, Hagerman PJ (2003). The (CGG)n repeat element within the 5′ untranslated region of the FMR1 message provides both positive and negative cis effects on in vivo translation of a downstream reporter.. Hum Mol Genet.

[9] Brouwer JR, Huizer K, Severijnen LA, Hukema RK, Berman RF (2008). CGG-repeat length and neuropathological and molecular correlates in a mouse model for fragile X-associated tremor/ataxia syndrome.. J Neurochem.

[10] Brouwer JR, Mientjes EJ, Bakker CE, Nieuwenhuizen IM, Severijnen LA (2007). Elevated Fmr1 mRNA levels and reduced protein expression in a mouse model with an unmethylated Fragile X full mutation.. Exp Cell Res.

[11] Willemsen R, Hoogeveen-Westerveld M, Reis S, Holstege J, Severijnen LA (2003). The FMR1 CGG repeat mouse displays ubiquitin-positive intranuclear neuronal inclusions; implications for the cerebellar tremor/ataxia syndrome.. Hum Mol Genet.

[12] Peprah E, He W, Allen E, Oliver T, Boyne A (2010). Examination of FMR1 transcript and protein levels among 74 premutation carriers.. J Hum Genet.

[13] Jin P, Zarnescu DC, Zhang F, Pearson CE, Lucchesi JC (2003). RNA-mediated neurodegeneration caused by the fragile X premutation rCGG repeats in Drosophila.. Neuron.

[14] Arocena DG, Iwahashi CK, Won N, Beilina A, Ludwig AL (2005). Induction of inclusion formation and disruption of lamin A/C structure by premutation CGG-repeat RNA in human cultured neural cells.. Hum Mol Genet.

[15] Handa V, Goldwater D, Stiles D, Cam M, Poy G (2005). Long CGG-repeat tracts are toxic to human cells: implications for carriers of Fragile X premutation alleles.. FEBS Lett.

[16] Hashem V, Galloway JN, Mori M, Willemsen R, Oostra BA (2009). Ectopic expression of CGG containing mRNA is neurotoxic in mammals.. Hum Mol Genet.

[17] Iwahashi CK, Yasui DH, An HJ, Greco CM, Tassone F (2006). Protein composition of the intranuclear inclusions of FXTAS.. Brain.

[18] Jin P, Duan R, Qurashi A, Qin Y, Tian D (2007). Pur alpha binds to rCGG repeats and modulates repeat-mediated neurodegeneration in a Drosophila model of fragile X tremor/ataxia syndrome.. Neuron.

[19] Sofola OA, Jin P, Qin Y, Duan R, Liu H (2007). RNA-binding proteins hnRNP A2/B1 and CUGBP1 suppress fragile X CGG premutation repeat-induced neurodegeneration in a Drosophila model of FXTAS.. Neuron.

[20] Sellier C, Rau F, Liu Y, Tassone F, Hukema RK (2010). Sam68 sequestration and partial loss of function are associated with splicing alterations in FXTAS patients.. EMBO J.

[21] Zang JB, Nosyreva ED, Spencer CM, Volk LJ, Musunuru K (2009). A mouse model of the human Fragile X syndrome I304N mutation.. PLoS Genet.

[22] Pietrobono R, Tabolacci E, Zalfa F, Zito I, Terracciano A (2005). Molecular dissection of the events leading to inactivation of the FMR1 gene.. Hum Mol Genet.

[23] Tabolacci E, Moscato U, Zalfa F, Bagni C, Chiurazzi P (2008). Epigenetic analysis reveals a euchromatic configuration in the FMR1 unmethylated full mutations.. Eur J Hum Genet.

[24] Feng Y, Zhang F, Lokey LK, Chastain JL, Lakkis L (1995). Translational suppression by trinucleotide repeat expansion at FMR1.. Science.

[25] Tassone F, Beilina A, Carosi C, Albertosi S, Bagni C (2007). Elevated FMR1 mRNA in premutation carriers is due to increased transcription.. RNA.

[26] Mulvihill DJ, Nichol Edamura K, Hagerman KA, Pearson CE, Wang YH (2005). Effect of CAT or AGG interruptions and CpG methylation on nucleosome assembly upon trinucleotide repeats on spinocerebellar ataxia, type 1 and fragile X syndrome.. J Biol Chem.

[27] Wang YH, Gellibolian R, Shimizu M, Wells RD, Griffith J (1996). Long CCG triplet repeat blocks exclude nucleosomes: a possible mechanism for the nature of fragile sites in chromosomes.. J Mol Biol.

[28] Cho Y, Griswold A, Campbell C, Min KT (2005). Individual histone deacetylases in Drosophila modulate transcription of distinct genes.. Genomics.

[29] Nedelsky NB, Todd PK, Taylor JP (2008). Autophagy and the ubiquitin-proteasome system: collaborators in neuroprotection.. Biochim Biophys Acta.

[30] Pandey UB, Nie Z, Batlevi Y, McCray BA, Ritson GP (2007). HDAC6 rescues neurodegeneration and provides an essential link between autophagy and the UPS.. Nature.

[31] Steffan JS, Bodai L, Pallos J, Poelman M, McCampbell A (2001). Histone deacetylase inhibitors arrest polyglutamine-dependent neurodegeneration in Drosophila.. Nature.

[32] Barlow AL, van Drunen CM, Johnson CA, Tweedie S, Bird A (2001). dSIR2 and dHDAC6: two novel, inhibitor-resistant deacetylases in Drosophila melanogaster.. Exp Cell Res.

[33] Du G, Liu X, Chen X, Song M, Yan Y Drosophila histone deacetylase 6 protects dopaminergic neurons against {alpha}-synuclein toxicity by promoting inclusion formation.. Mol Biol Cell.

[34] Pallos J, Bodai L, Lukacsovich T, Purcell JM, Steffan JS (2008). Inhibition of specific HDACs and sirtuins suppresses pathogenesis in a Drosophila model of Huntington's disease.. Hum Mol Genet.

[35] Strahl BD, Allis CD (2000). The language of covalent histone modifications.. Nature.

[36] Coffee B, Zhang F, Ceman S, Warren ST, Reines D (2002). Histone modifications depict an aberrantly heterochromatinized FMR1 gene in fragile x syndrome.. Am J Hum Genet.

[37] Coffee B, Zhang F, Warren ST, Reines D (1999). Acetylated histones are associated with FMR1 in normal but not fragile X-syndrome cells.. Nat Genet.

[38] Biacsi R, Kumari D, Usdin K (2008). SIRT1 inhibition alleviates gene silencing in Fragile X mental retardation syndrome.. PLoS Genet.

[39] Habib M, Fares F, Bourgeois CA, Bella C, Bernardino J (1999). DNA global hypomethylation in EBV-transformed interphase nuclei.. Exp Cell Res.

[40] Brennan EP, Ehrich M, Brazil DP, Crean JK, Murphy M (2009). Comparative analysis of DNA methylation profiles in peripheral blood leukocytes versus lymphoblastoid cell lines.. Epigenetics.

[41] Grafodatskaya D, Choufani S, Ferreira JC, Butcher DT, Lou Y EBV transformation and cell culturing destabilizes DNA methylation in human lymphoblastoid cell lines.. Genomics.

[42] Garcia-Arocena D, Yang JE, Brouwer JR, Tassone F, Iwahashi C Fibroblast phenotype in male carriers of FMR1 premutation alleles.. Hum Mol Genet.

[43] Shahbazian MD, Grunstein M (2007). Functions of site-specific histone acetylation and deacetylation.. Annu Rev Biochem.

[44] Balasubramanyam K, Altaf M, Varier RA, Swaminathan V, Ravindran A (2004). Polyisoprenylated benzophenone, garcinol, a natural histone acetyltransferase inhibitor, represses chromatin transcription and alters global gene expression.. J Biol Chem.

[45] Balasubramanyam K, Swaminathan V, Ranganathan A, Kundu TK (2003). Small molecule modulators of histone acetyltransferase p300.. J Biol Chem.

[46] Pan MH, Chang WL, Lin-Shiau SY, Ho CT, Lin JK (2001). Induction of apoptosis by garcinol and curcumin through cytochrome c release and activation of caspases in human leukemia HL-60 cells.. J Agric Food Chem.

[47] Osterwalder T, Yoon KS, White BH, Keshishian H (2001). A conditional tissue-specific transgene expression system using inducible GAL4.. Proc Natl Acad Sci U S A.

[48] Entezam A, Biacsi R, Orrison B, Saha T, Hoffman GE (2007). Regional FMRP deficits and large repeat expansions into the full mutation range in a new Fragile X premutation mouse model.. Gene.

[49] Usdin K (2008). The biological effects of simple tandem repeats: lessons from the repeat expansion diseases.. Genome Res.

[50] Thomson JP, Skene PJ, Selfridge J, Clouaire T, Guy J CpG islands influence chromatin structure via the CpG-binding protein Cfp1.. Nature.

[51] Sutcliffe JS, Nelson DL, Zhang F, Pieretti M, Caskey CT (1992). DNA methylation represses FMR-1 transcription in fragile X syndrome.. Hum Mol Genet.

[52] Hornstra IK, Nelson DL, Warren ST, Yang TP (1993). High resolution methylation analysis of the FMR1 gene trinucleotide repeat region in fragile X syndrome.. Hum Mol Genet.

[53] Tabolacci E, De Pascalis I, Accadia M, Terracciano A, Moscato U (2008). Modest reactivation of the mutant FMR1 gene by valproic acid is accompanied by histone modifications but not DNA demethylation.. Pharmacogenet Genomics.

[54] Torrioli M, Vernacotola S, Setini C, Bevilacqua F, Martinelli D Treatment with valproic acid ameliorates ADHD symptoms in fragile X syndrome boys.. Am J Med Genet A.

[55] Tabolacci E, Pietrobono R, Moscato U, Oostra BA, Chiurazzi P (2005). Differential epigenetic modifications in the FMR1 gene of the fragile X syndrome after reactivating pharmacological treatments.. Eur J Hum Genet.

[56] Tassone F, Hagerman RJ, Chamberlain WD, Hagerman PJ (2000). Transcription of the FMR1 gene in individuals with fragile X syndrome.. Am J Med Genet.

[57] Greco CM, Soontrapornchai K, Wirojanan J, Gould JE, Hagerman PJ (2007). Testicular and pituitary inclusion formation in fragile X associated tremor/ataxia syndrome.. J Urol.

[58] Rodriguez-Revenga L, Madrigal I, Pagonabarraga J, Xuncla M, Badenas C (2009). Penetrance of FMR1 premutation associated pathologies in fragile X syndrome families.. Eur J Hum Genet.

[59] Ladd PD, Smith LE, Rabaia NA, Moore JM, Georges SA (2007). An antisense transcript spanning the CGG repeat region of FMR1 is upregulated in premutation carriers but silenced in full mutation individuals.. Hum Mol Genet.

[60] Sofola OA, Jin P, Botas J, Nelson DL (2007). Argonaute-2-dependent rescue of a Drosophila model of FXTAS by FRAXE premutation repeat.. Hum Mol Genet.

[61] Liao CH, Sang S, Ho CT, Lin JK (2005). Garcinol modulates tyrosine phosphorylation of FAK and subsequently induces apoptosis through down-regulation of Src, ERK, and Akt survival signaling in human colon cancer cells.. J Cell Biochem.

[62] Ito C, Itoigawa M, Miyamoto Y, Onoda S, Rao KS (2003). Polyprenylated benzophenones from Garcinia assigu and their potential cancer chemopreventive activities.. J Nat Prod.

[63] Acevedo HR, Rojas MD, Arceo SD, Soto Hernandez M, Martinez Vazquez M (2006). Effect of 6-nonadecyl salicylic acid and its methyl ester on the induction of micronuclei in polychromatic erythrocytes in mouse peripheral blood.. Mutat Res.

[64] Tanaka T, Kohno H, Shimada R, Kagami S, Yamaguchi F (2000). Prevention of colonic aberrant crypt foci by dietary feeding of garcinol in male F344 rats.. Carcinogenesis.

[65] Chong SS, Eichler EE, Nelson DL, Hughes MR (1994). Robust amplification and ethidium-visible detection of the fragile X syndrome CGG repeat using Pfu polymerase.. Am J Med Genet.

[66] Kramer PR, Pearson CE, Sinden RR (1996). Stability of triplet repeats of myotonic dystrophy and fragile X loci in human mutator mismatch repair cell lines.. Hum Genet.

[67] Tassone F, Pan R, Amiri K, Taylor AK, Hagerman PJ (2008). A rapid polymerase chain reaction-based screening method for identification of all expanded alleles of the fragile X (FMR1) gene in newborn and high-risk populations.. J Mol Diagn.

[68] Kazemi-Esfarjani P, Benzer S (2000). Genetic suppression of polyglutamine toxicity in Drosophila.. Science.

[69] Rodriguez-Lebron E, Gouvion CM, Moore SA, Davidson BL, Paulson HL (2009). Allele-specific RNAi mitigates phenotypic progression in a transgenic model of Alzheimer's disease.. Mol Ther.

[70] Berry-Kravis E, Abrams L, Coffey SM, Hall DA, Greco C (2007). Fragile X-associated tremor/ataxia syndrome: clinical features, genetics, and testing guidelines.. Mov Disord.

[71] Patel SR, Kim D, Levitan I, Dressler GR (2007). The BRCT-domain containing protein PTIP links PAX2 to a histone H3, lysine 4 methyltransferase complex.. Dev Cell.

[72] McGuire SE, Mao Z, Davis RL (2004). Spatiotemporal gene expression targeting with the TARGET and gene-switch systems in Drosophila.. Sci STKE.

[73] Todd PK, Mack KJ (2000). Sensory stimulation increases cortical expression of the fragile X mental retardation protein in vivo.. Brain Res Mol Brain Res.

