# Supplementary figures and images for: Histone Deacetylases Suppress CGG Repeat–Induced Neurodegeneration Via Transcriptional Silencing in Models of Fragile X Tremor Ataxia Syndrome
Source: PLoS Genet. 2010 Dec 9;6(12):e1001240. doi: 10.1371/journal.pgen.1001240 (PMC3000359; doi:10.1371/journal.pgen.1001240)

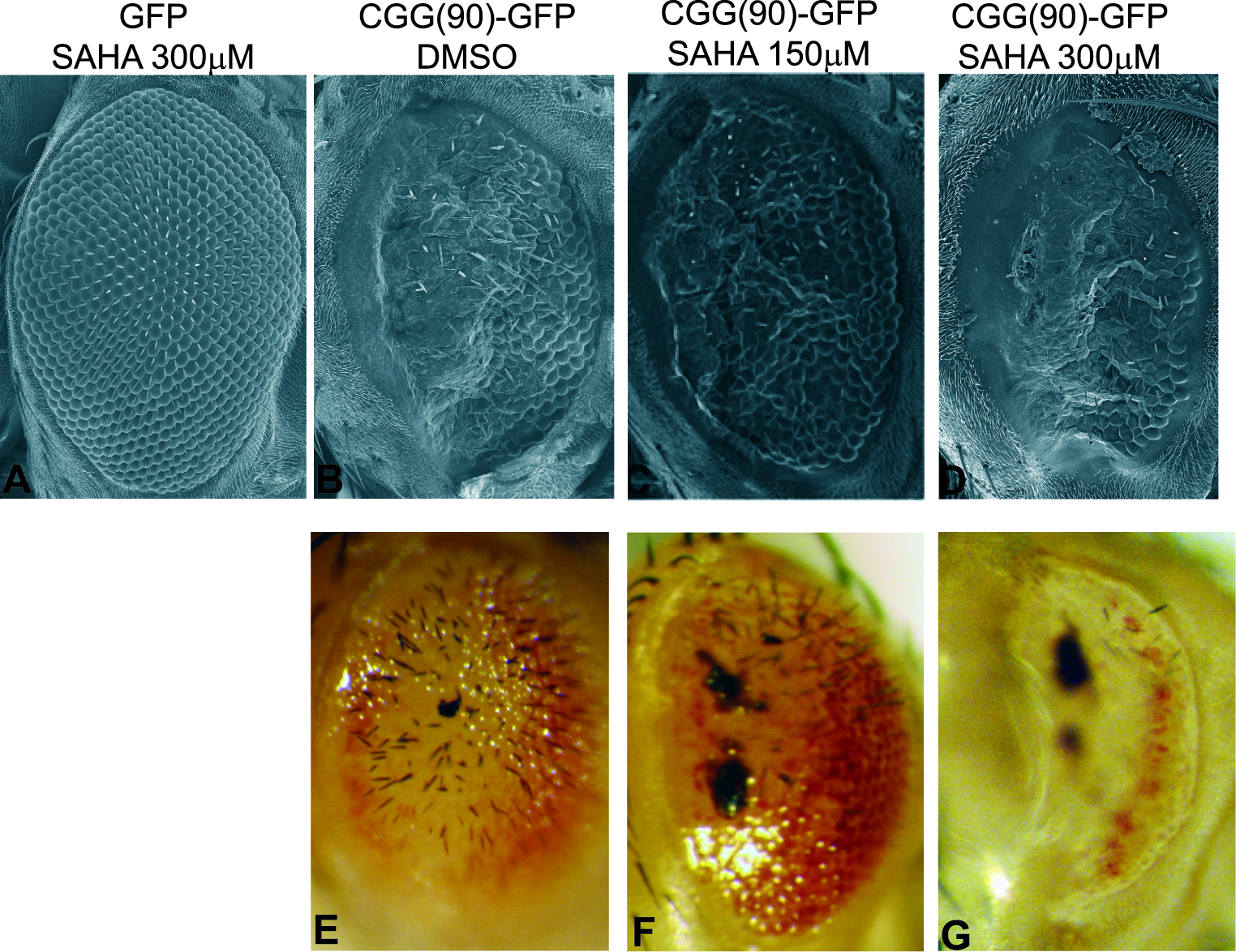

Supplement: Figure S1 — The HDAC inhibitor SAHA exacerbates CGG repeat dependent neurodegeneration. Scanning electron microscopy (A–D) or light microscopy (E–G) images were obtained from flies expressing eGFP (A) or (CGG)90-eGFP line I (B–G). Flies were reared at 25C in standard fly food containing either DMSO (B,E) or the broad spectrum HDAC inhibitor SAHA at 150 µM (C, F) or 300 µM (A,D,G). Images are representative of greater than 25 flies per treatment group. (3.86 MB TIF) [file pgen.1001240.s001.tif]

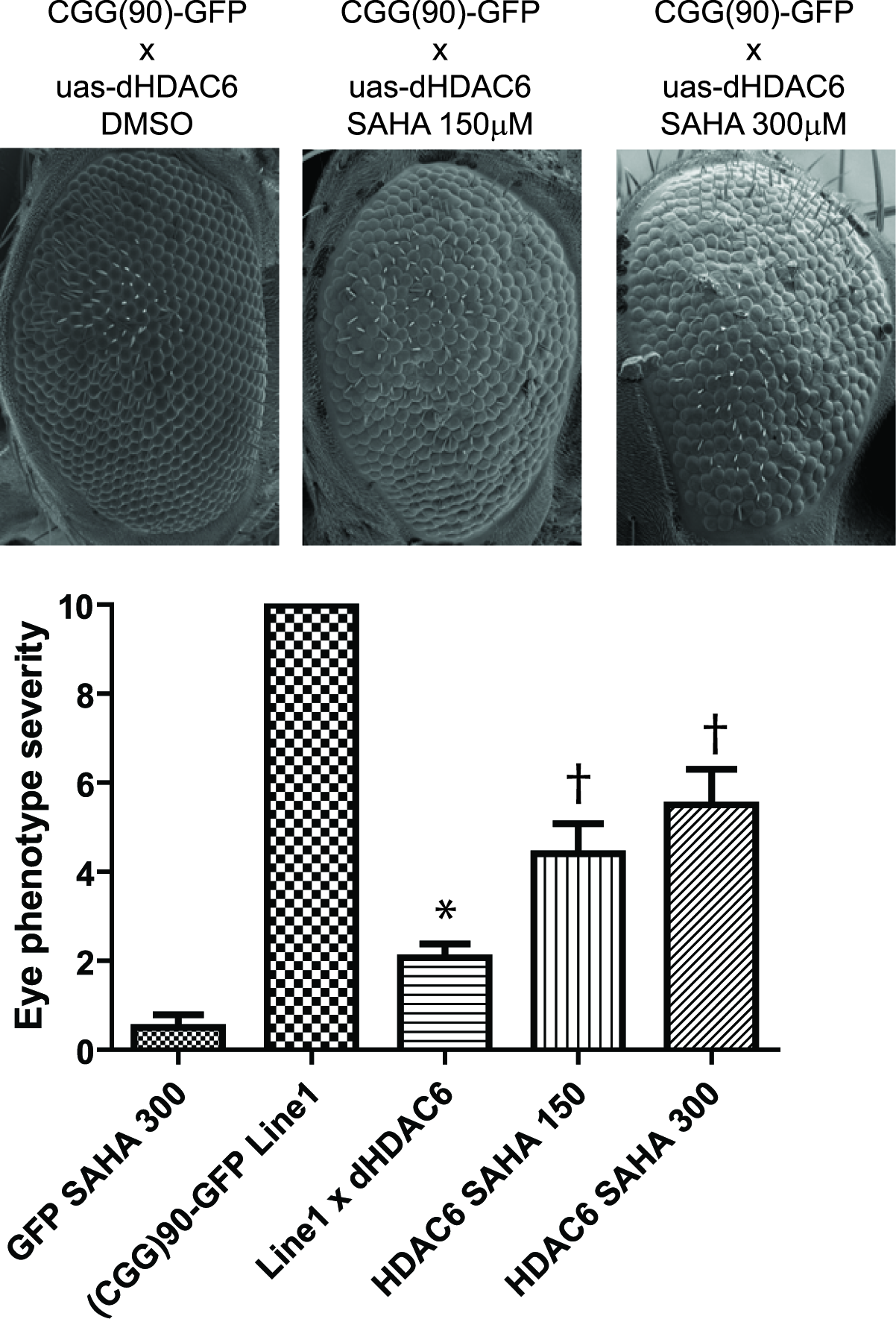

Supplement: Figure S2 — The HDAC inhibitor SAHA partially suppresses rescue by dHDAC6 of CGG repeat dependent neurodegeneration. Representative SEM images from drosophila co-expressing (CGG)90-eGFP line I and UAS- dHDAC6 reared on DMSO(A), or SAHA at 150 µM (B) or 300 µM (C). Quantitation of multiple flies reveals a significant worsening of the phenotype in flies reared on SAHA. (* = p<0.01 compared to (CGG)90-eGFP line I alone, + = p<0.01 compared to (CGG)90-eGFP line I x dHDAC6 by a Students unpaired t-test). (1.38 MB TIF) [file pgen.1001240.s002.tif]

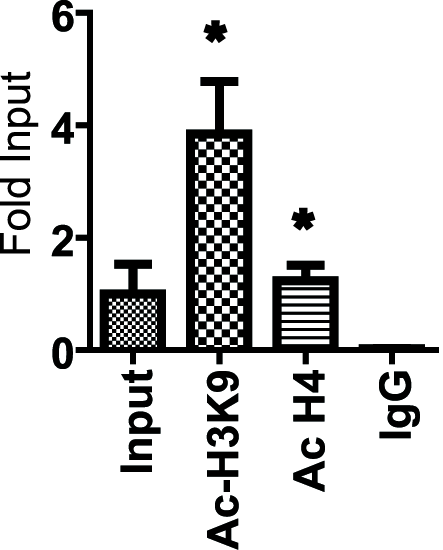

Supplement: Figure S3 — Chromatin immunoprecipitation from pre-mutation carrier cells. Input is crosslink reversed DNA not subjected to immunoprecipitation. Data are expressed as a ratio to the FMR1 exon 1 signal from the input material. There is enrichment of the FMR1 exon 1 locus in pre-mutation carrier lymphoblast cell lines when ChIP is performed against either Ac-histone H3K9 or Ac-histone H4 compared to IgG alone. * = P<0.001 by unpaired t-test compared to IgG immunoprecipitation alone. (0.05 MB TIF) [file pgen.1001240.s003.tif]

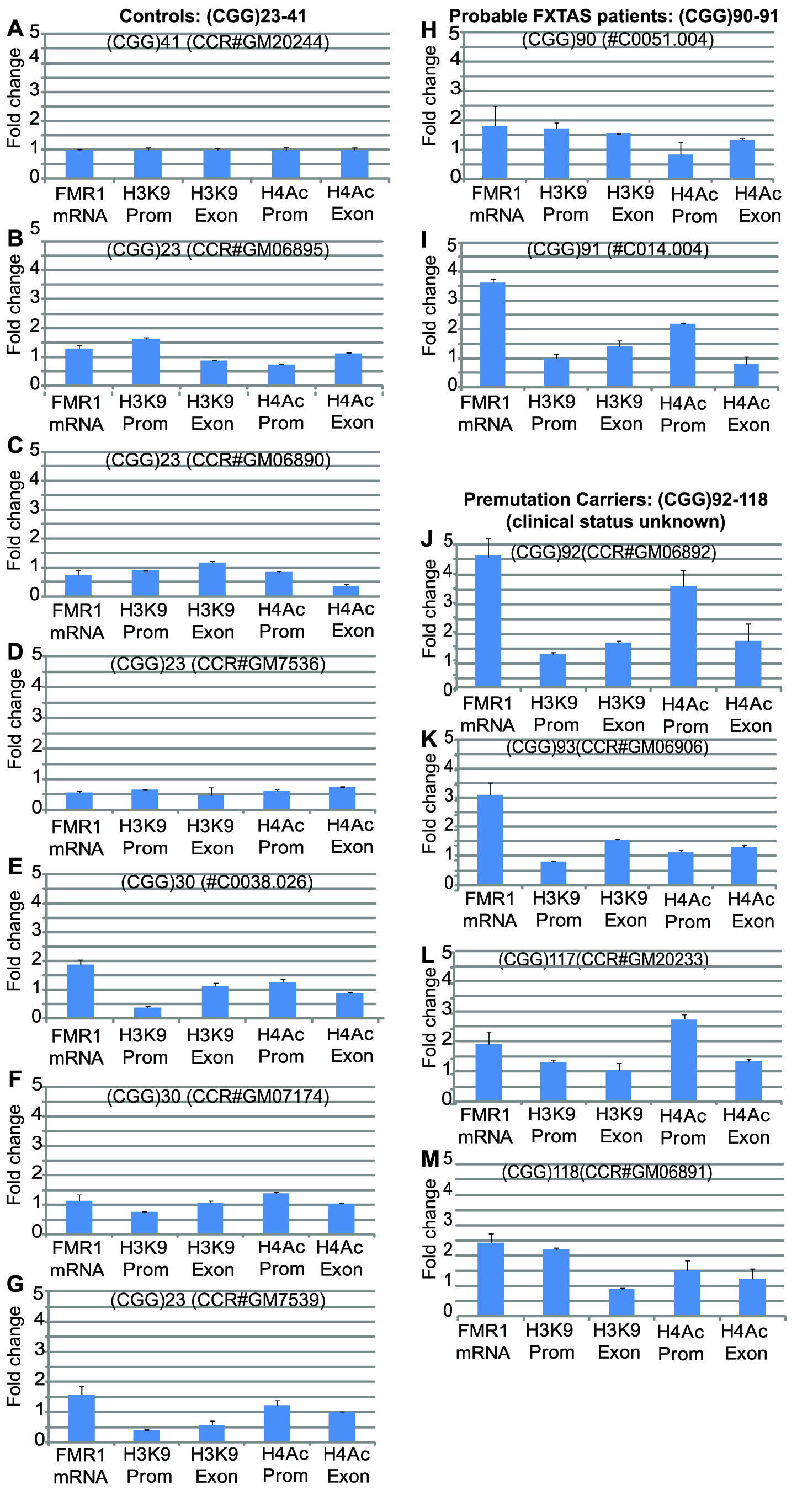

Supplement: Figure S4 — Chromatin immunoprecipitation results from each lymphoblast cell line. ChIP was performed 3–4 times on each of 13 different lymphoblastoid cell lines. The data from each cell line is provided. FMR1 mRNA and ChIP results for all samples were normalized to cell line GM20244 (CGG)41 during each qPCR run to allow for run to run comparisons. Cell lines are divided into normal repeat lengths (A–G), Clinically probable FXTAS patient-derived cell lines (H,I), and pre-mutation carrier derived cell lines whose clinical status is unknown (J–M). (0.50 MB TIF) [file pgen.1001240.s004.tif]

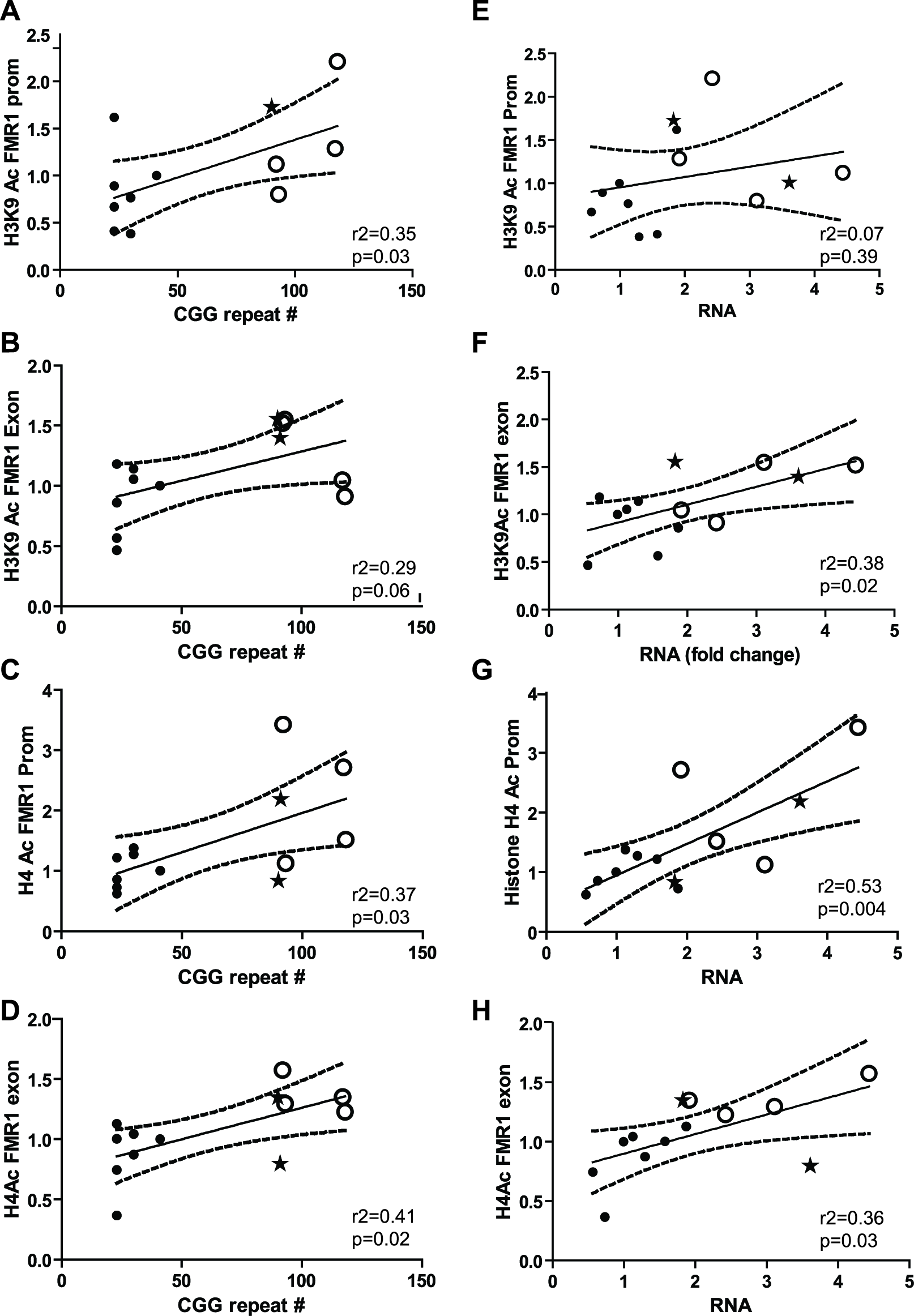

Supplement: Figure S5 — Correlations between ChIP AcH3K9 and AcH4 to CGG repeat number and FMR1 mRNA expression. For each graph, solid black dots = control cell lines, stars = confirmed FXTAS cases, open circles = pre-mutation carriers whose clinical status is unknown. The central line is the linear best fit. Curved dashed lines are 95% confidence intervals. The r2 and significance for each correlation is shown in each graph. ChIP to Ac H3K9 correlated with CGG repeat number using PCR primers directed at either the FMR1 promoter (Fig A, FMR1 prom AcH3K9 to CGG#) or the FMR1 exon (Fig B, FMR1 exon AcH3K9 to CGG#). Correlation of ChIP to Ac H3K9 and FMR1 mRNA expression was significant using PCR primers directed at the FMR1 exon (Fig F, FMR1 exon H3K9 to FMR1 mRNA), but not the FMR1 promoter (Fig E, FMR1 prom AcH3K9 to FMR1 mRNA). ChIP against Ac H4 correlated with CGG repeat number (Fig C,FMR1 prom AcH4 to CGG#; Fig D, FMR1 exon H4 to CGG#) and FMR1 mRNA expression (Fig G, FMR1 prom AcH4 to FMR1 mRNA; Fig H, FMR1 exon AcH4 to FMR1 mRNA) using PCR primers directed at either the FMR1 promoter or the FMR1 first exon. (0.67 MB TIF) [file pgen.1001240.s005.tif]

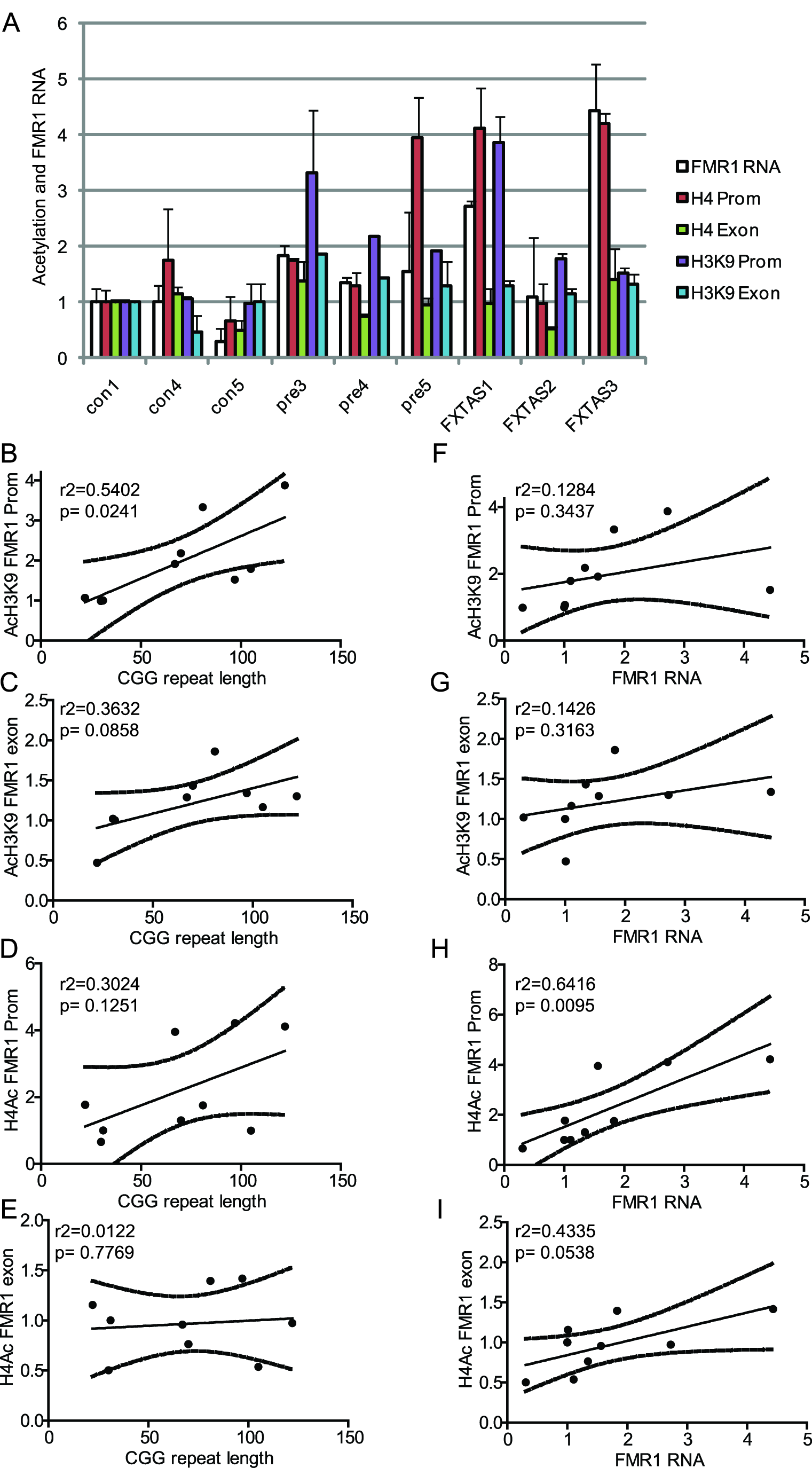

Supplement: Figure S6 — ChIP and FMR1 mRNA results from individual fibroblast cell lines. A) ChIP against Ac H3K9 or pan acetylated H4 and FMR1 mRNA expression normalized to Actin mRNA expression is shown for each sell line. All data is presented as fold change from Control fibroblast line #C1. Error bars represent SD from 2–3 independent experiments. B–E) Correlations of individual acetylated chromatin marks as determined by ChIP (y-axis) with CGG repeat number (x-axis). F–I) Correlation between individual Acetylated Chromatin marks as determined by ChIP (y-axis) with FMR1 mRNA expression (x-axis). For each, r2 and significance of Pearson correlation is provided as an inset. (0.44 MB TIF) [file pgen.1001240.s006.tif]

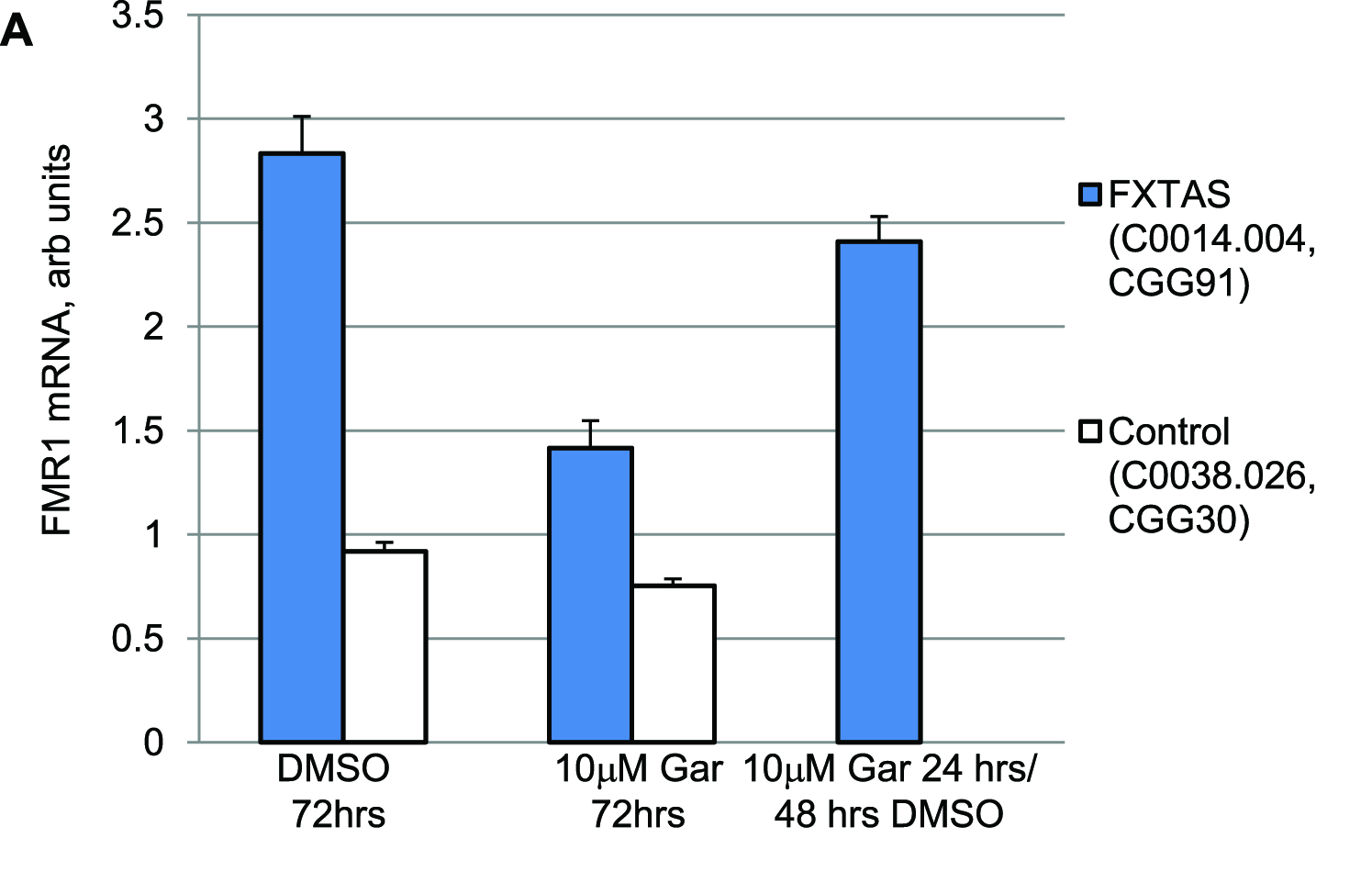

Supplement: Figure S7 — Garcinol effects on FMR1 expression are transient. Lymphoblasts derived from a patient with probable FXTAS (#C0014.004, CGG91 repeats) or from a control patient were treated for 24 or 72 hours with 10 µM garcinol or DMSO. After 24 hours, some Garcinol treated cells had their media changed to include only DMSO for 48 hours. Equal numbers of cells were harvested and mRNA was extracted and quantified by qPCR. FMR1 mRNA levels are normalized to 18S mRNA and expressed (approximately) as a ratio to FMR1 expression in DMSO treated cells. There is a significant reduction in FMR1 mRNA expression in FXTAS cells treated for 72 hours with Garcinol, but there is no significant difference in FMR1 expression in cells treated with Garcinol for only 24 hours and then switched to vehicle. *P = 0.05, Students t-test versus DMSO treated cells. (0.12 MB TIF) [file pgen.1001240.s007.tif]

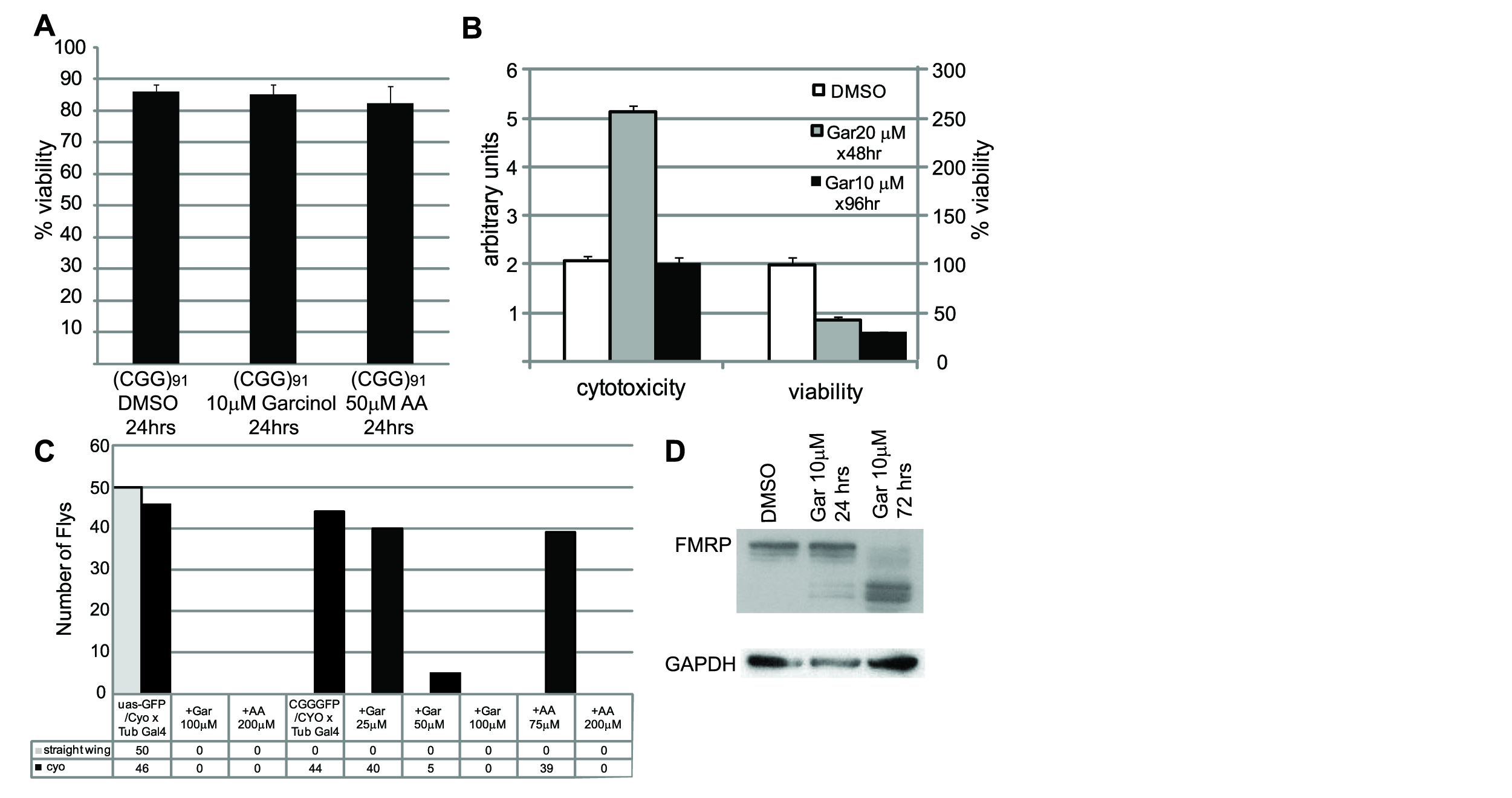

Supplement: Figure S8 — Toxic effects of HAT inhibitors on lymphoblast cells and fly eclosion. Exposure to HAT inhibitors has been reported as toxic to cancer cells. We therefore assessed the effects on viability and cytotoxicity of various doses of HAT inhibitors on lymphoblast cell lines. A) Treatment for 24 hours with garcinol (10 µM) or anacardic acid (50 µM) at the minimally effective dose for altering FMR1 mRNA expression did not alter cell viability. B) However, at higher doses (20–50 µM garcinol) and with longer exposures (48–96 hrs), these drugs were toxic to lymphoblast cell lines. C) These drugs also blocked eclosion of flies reared on doses greater than 25 µM. Garcinol or 75 µM anacardic acid, which precluded performing some phenotypic rescue experiments. D) Consistent with the decrease seen in FMR1 mRNA expression, FMRP levels are stable after 24 hours of exposure to10 µM garcinol but are significantly depressed after 72 hours of exposure, although interpretation of this later time point may be complicated by decreased cell viability (note lower molecular weight degradation products of FMRP at this time point). (0.30 MB TIF) [file pgen.1001240.s008.tif]
